# Interrogation of human microglial phagocytosis by CRISPR genome editing

**DOI:** 10.3389/fimmu.2023.1169725

**Published:** 2023-07-07

**Authors:** Jason Cheng-Yu Chang, Cheng-You Wang, Steven Lin

**Affiliations:** ^1^ Institute of Biological Chemistry, Academia Sinica, Taipei, Taiwan; ^2^ Institute of Biochemical Sciences, National Taiwan University, Taipei, Taiwan

**Keywords:** microglia, CRISPR, Cas9 RNP, phagocytosis, amyloid beta, glioblastoma, HMC3 and BV-2

## Abstract

**Background:**

Microglia are an integral part of central nervous system, but our understanding of microglial biology is limited due to the challenges in obtaining and culturing primary human microglia. HMC3 is an important cell line for studying human microglia because it is readily accessible and straightforward to maintain in standard laboratories. Although HMC3 is widely used for microglial research, a robust genetic method has not been described. Here, we report a CRISPR genome editing platform, by the electroporation of Cas9 ribonucleoproteins (Cas9 RNP) and synthetic DNA repair templates, to enable rapid and precise genetic modifications of HMC3. For proof-of-concept demonstrations, we targeted the genes implicated in the regulation of amyloid beta (Aβ) and glioblastoma phagocytosis in microglia. We showed that CRISPR genome editing could enhance the phagocytic activities of HMC3.

**Methods:**

We performed CRISPR gene knockout (KO) in HMC3 by the electroporation of pre-assembled Cas9 RNP. Co-introduction of DNA repair templates allowed site-specific knock-in (KI) of an epitope tag, a synthetic promoter and a fluorescent reporter gene. The editing efficiencies were determined genotypically by DNA sequencing and phenotypically by immunofluorescent staining and flow cytometry. The gene-edited HMC3 cells were examined *in vitro* by fluorescent Aβ and glioblastoma phagocytosis assays.

**Results:**

Our platform enabled robust single (>90%) and double (>70%) KO without detectable off-target editing by high throughput DNA sequencing. We also inserted a synthetic SFFV promoter to efficiently upregulate the expression of endogenous *CD14* and *TREM2* genes associated with microglial phagocytosis. The CRISPR-edited HMC3 showed stable phenotypes and enhanced phagocytosis of fluorescence-labeled Aβ1-42 peptides. Confocal microscopy further confirmed the localization of Aβ_1-42_ aggregates in the acidified lysosomes. HMC3 mutants also changed the phagocytic characteristic toward apoptotic glioblastoma cells.

**Conclusion:**

CRISPR genome editing by Cas9 RNP electroporation is a robust approach to genetically modify HMC3 for functional studies such as the interrogation of Aβ and tumor phagocytosis, and is readily adoptable to investigate other aspects of microglial biology.

## Introduction

Microglia are resident immune cells of the central nervous system (CNS) and play vital roles in brain development, homeostasis and immune surveillance (1, 2). Microglia originate from erythro-myeloid progenitors in the yolk sac during early embryonic stage, and maintain their population by self-renewal without the input from bone marrow-derived myeloid precursors (3). Microglia are widely distributed throughout the brain and constantly interact with neurons, astrocytes, and blood vessels via surface receptors to monitor the CNS microenvironment (2, 3). Upon activation by neuropathological stimuli, microglia phagocytose damaged cells and debris, and secret cytokines, neurotrophic factors and neuroprotective factors to orchestrate immune response and tissue repair in the CNS (2, 4). Aberrant microglial functions have been implicated in a myriad of neurogenesis defects, neurological diseases and brain tumors (1, 2, 5). Understanding the microglia-CNS interplay is therefore crucial to the maintenance of a healthy CNS and the treatment of brain diseases.

The advance in microglial research techniques and CNS disease models has revealed exciting new insights into microglial biology (6). However, many studies were conducted with murine and rodent microglia because primary human microglia are difficult to obtain and culture. Due to ethical reasons, the source of primary human microglia is restricted to aborted fetal tissues, biopsies from epileptic patients, brain tumor excision and postmortem brain tissue (7). To overcome this limitation, several microglial cell lines were generated to study human microglial functions and to validate the observations made in animal models. Human microglial clone 3 cell line (HMC3, also known as CHME-5) is the most studied and best characterized. HMC3 was established from human embryonic microglial cells by SV40-dependent immortalization (8), and was authenticated by the American Type Culture Collection (ATCC) in 2018 (7). HMC3 is highly similar to the primary cells in microglial markers and functions (7), and has been used to study many aspects of microglial biology such as inflammatory responses, phagocytosis and cell migration (9–16). Here, we report an CRISPR genome editing platform for HMC3 to facilitate genetic and functional investigation.

**Figure 1:**
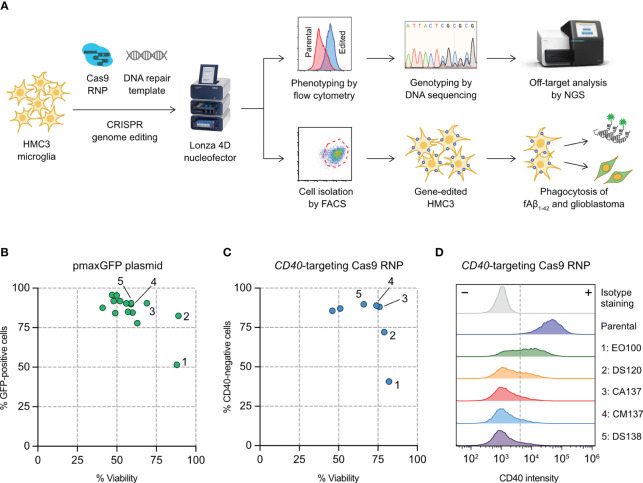
Optimization of HMC3 genome editing by Cas9 ribonucleoproteins (RNP) electroporation. **(A)** Cas9 RNP and DNA repair templates were electroporated into HMC3 cells by a Lonza 4D nucleofector system. The editing efficiency was determined genotypically by DNA sequencing and ICE analysis, and phenotypically using immunofluorescent staining and flow cytometry. Off-target editing was analyzed by next generation sequencing (NGS). Gene-edited cells were isolated by fluorescence-activated cell sorting (FACS), and assayed for *in vitro* phagocytosis of fluorescently labeled amyloid beta peptide (fAβ_1-42_). **(B)** Screening of electroporation pulse codes for the optimal DNA delivery using plasmid pmaxGFP encoding the *turboGFP* gene. **(C)** Screening of pulse codes for Cas9 RNP delivery using *CD40*-targeting Cas9. **(D)** Shift in CD40 intensity after Cas9 RNP electroporation by the indicated pulse codes. The complete list of pulse codes is in **Table S3**.

CRISPR gene KO has been reported in mouse primary microglia, induced pluripotent stem cell-differentiated microglia, and microglial cell lines by the conventional plasmid- and lentiviral vector-based procedures (9, 17–22). In this work, we report a different approach for CRISPR gene KO and KI by the electroporation of pre-assembled Cas9 ribonucleoproteins (RNP, **Figure 1A**). Gene editing by Cas9 RNP electroporation is a popular method for human cells because of rapid editing action, high precision, low toxicity and free of foreign genetic materials such as plasmid and viral DNA (23–25). In comparison to the gesicle-mediated Cas9 RNP for gene KO in CHME-5 (26), electroporation is more straightforward to perform. Through systematic optimization, we achieved highly efficient single and double gene KO (up to 90%) at multiple loci across the genome. Furthermore, co-electroporation of synthetic DNA repair templates allowed site-specific DNA modifications and gene KI via homology-directed repair (HDR) at Cas9-induced DNA double-strand breaks (DSB). We successfully created precise, in-frame insertion of a hemagglutinin (HA) epitope tag, a *gfp* reporter gene, and a synthetic promoter sequence to upregulate the expression of endogenous genes. The gene-edited cells were isolated and expanded to stable and near homogenous populations for functional studies.

To show the versatility of our gene-editing platform, we edited the *CD14*, *CD47*, *TREM2, SIRPA* and *MERTK* genes implicated in the clearance of Aβ and glioblastoma cells. We performed *in vitro* assays to measure the phagocytosis of fluorescence-labeled amyloid beta (fAβ_1-42_) aggregates in the gene-edited HMC3 cells. We show that Aβ phagocytosis could be enhanced by increasing the expression of TREM2 and CD14 receptors. Also, the TREM2-positive mutant demonstrated increased phagocytosis toward both live and apoptotic glioblastoma cells. Our Cas9 RNP editing platform and the collection of HMC3 mutant cells can help delineate the complex phagocytotic mechanisms underlying Alzheimer’s disease (AD) and glioblastoma, and can be readily applied to study other aspects of microglial biology.

## Materials and methods

### Reagents and antibodies

All chemicals were purchased from Sigma-Aldrich, cell culture media and reagents from Thermo Fisher Scientific and antibodies from BioLegend, unless specified otherwise. The following antibodies were used for flow cytometry analysis: PE anti-CD11b (ICRF44), PE anti-CD14 (63D3), PE anti-CD16 (3G8), PE anti-CD22 (S-HCL-1), APC anti-CD33 (P67.6), APC anti-CD36 (5-271), APC anti-CD40 (5C3), APC anti-CD45 (2D1), APC anti-CD47 (CC2C6), FITC anti-CD64 (10.1), APC anti-CD68 (Y1/82A), APC anti-CD96 (NK92.39), FITC anti-CD206 (15-2), PE anti-CX3CR1 (2A9-1), PE anti-HLA-DR (L243), PE anti-IL-4Rα (G077F6), PE anti-LILRB2 (42D1), PE anti-MERTK (590H11G1E3), APC anti-PD1 (EH12.2H7), APC anti-PYRY12 (S16001E), PE anti-Siglec10 (5G6), APC anti-SIRPα (SE5A5), PE anti-SIRPα (SE5A5), PE anti-TGFBRII (REA903, Miltenyi Biotec), PE anti-Tim3 (A18087E), APC anti-TREM2 (# 237920, R&D Systems), APC Mouse IgG1κ Isotype (MOPC-21), APC Mouse IgG2aκ Isotype (MOPC-173), APC Mouse IgG2bκ Isotype (MOPC-11), PE Mouse IgG1κ Isotype (MOPC-21), PE Mouse IgG2aκ Isotype (MOPC-173), PE Mouse IgG2bκ Isotype (MOPC-11), PE Rat IgG2aκ Isotype (RTK2758), APC Rat IgG2B Isotype (# 141945, R&D Systems), and APC anti-HA.11 Epitope-Tag (16B12). For BV-2 mouse microglia: APC anti-mouse CD40 (3/23) and APC Rat IgG2aκ Isotype (RTK2758).

### Cell culture

Human microglia HMC3 cell line was purchased from ATCC (catalog # CRL-3304) and cultured in Dulbecco’s modified Eagle medium: Nutrient Mixture F-12 (Hyclone) supplemented with 10% heat-inactivated fetal bovine serum (FBS), 1X penicillin/streptomycin and 10 mM HEPES at 37°C in a humidified incubator containing 5% CO_2_. The complete medium is thereafter abbreviated as DMEM/F12. The cells were passaged at 80% confluency. The cells were rinsed with DPBS (Corning), and treated with Accutase (Thermo Fisher Scientific) for 5 min. After dissociation, pre-warmed DMEM/F12 was added to adjust the cell density to 3-5 × 10^5^ cells per 10-cm culture dish. Mouse microglia BV-2 cell line was a kind gift from Dr. Yijuang Chern at Institute of Biomedical Sciences in Academia Sinica, Taiwan. BV-2 was cultured in DMEM/F12 supplemented with 1X Glutamax (Thermo Fisher Scientific) by the same procedure as HMC3. LN-229 glioblastoma cell line was purchased from ATCC (catalog # CRL-2611) and maintained per ATCC’s protocol in Dulbecco’s modified Eagle medium (Hyclone) supplemented with 10% heat-inactivated FBS, 1X penicillin/streptomycin and 10 mM HEPES at 37°C in a humidified incubator containing 5% CO_2_. All cell lines and the gene-edited cells were monitored routinely for mycoplasma contamination by Mycoplasma PCR Detection Kit (Biosmart). Cell density and viability during routine cell culture were determined by Trypan blue staining in Countess II cell counter (Thermo Fisher Scientific).

### Cas9 RNP preparation

Recombinant *Streptococcus pyogenes* Cas9 protein and synthetic CRISPR single guide RNA (sgRNA) were prepared as described in Lin et al., 2022 (27). Briefly, Cas9 protein was expressed from plasmid pSL103 (Addgene #182032) in *Escherichia coli* Tuner DE3 strain (Novagen). The protein was purified sequentially by Ni-NTA nickel-affinity column (Qiagen), Heparin ion-exchange column (Cytiva) and Superdex 200 gel filtration column (Cytiva). Cas9 protein was adjusted to 40 μM in 20 mM HEPES (pH 7.5), 150 mM KCl, β-mercaptoethanol and 10% (v/v) glycerol, and stored at −80°C. The sgRNA was designed *in silico* using CRISPR Design Tool on the Benchling website (http://www.benchling.com). The sgRNA was synthesized by *in vitro* transcription using PCR-assembled DNA templates and T7 RNA polymerase (Roche). The sequences of sgRNA and DNA oligonucleotides for DNA template assembled are listed in **Table S1**. The synthesized sgRNA was purified by denaturing polyacrylamide gel electrophoresis containing 10% (w/v) urea to extract the full-length sgRNA. The sgRNA was then treated with calf intestinal alkaline phosphatase (NEB) to remove the 5’ triphosphate moiety to prevent the activation of innate anti-RNA immune response (28). The sgRNA concentration was determined by absorbance at OD_260nm_ using NanoDrop (Thermo Fisher Scientific), adjusted to 48 μM in 20 mM HEPES (pH 7.5), 150 mM KCl, 10% (v/v) glycerol, 1 mM β-mercaptoethanol, and 1 mM MgCl_2_, and stored at -80°C. Both Cas9 protein and sgRNA were thawed immediately before RNP formation and never re-frozen. Cas9 RNP was prepared for electroporation by mixing equal volumes of 40 μM of Cas9 protein and 48 μM sgRNA to a final molar ratio of 1:1.2. The resulting 20-μM Cas9 RNP was then incubated at 37°C for 10 min for complex formation and kept at room temperature before use for up to an hour.

### Construction of HDR templates

The HDR template sequences, PCR primers and thermocycler settings are listed in **Table S2**. All DNA products were generated using KAPA HiFi HotStart DNA Polymerase kit (KAPA Biosystems). The design of HDR templates consists of three DNA fragments: the 5’ homology arm (fragment 1), the gene insert (fragment 2) and the 3’ homology arm (fragment 3). The *ACTB*-HA and *RAB11A*-HA templates contained the HA tag sequence flanked by 90 nt of 5’ and 3’ homology arms, and were purchased as ready-to-use, single-strand ultramers from IDT-DNA. The *ACTB*-GFP and *RAB11A*-GFP HDR templates contained the *gfp* reporter sequence flanked by 300 nt of 5’ and 3’ homology arms. The linear HDR templates were PCR-amplified from plasmid AICSDP-15 (Addgene #87425) and pTR-143 (Addgene #112012), and purified by QIAquick PCR Purification Kit (Qiagen).

To construct the *CD14*-SFFV template, fragment 1 and 3 homology arms were PCR-amplified from the *CD14* gene from HMC3 genomic DNA. Fragment 2 (SFFV promoter sequence) was amplified from plasmid LeGO-iT2 (Addgene # 27343). The PCR products were gel purified and extracted by Zymoclean Gel DNA Recovery Kits (Zymo Research). The three fragments were cloned into pUC19 vector by NEBuilder HiFi DNA Assembly kit (NEB) and designated as pSL387 plasmid. The plasmid was extracted by ZR Plasmid Miniprep Classic Kit (Zymo Research), and validated by Sanger sequencing at the DNA sequencing core facility at the Institute of Biomedical Sciences, Academia Sinica. The *TREM2*-SFFV template was constructed similarly and designated as pSL430 plasmid. The linear HDR templates were PCR-amplified from the pSL387 and 430 plasmids, and purified by QIAquick PCR Purification Kit (Qiagen). All HDR templates were diluted in molecular H_2_O to 50 μM, and stored at -20°C.

### Electroporation of Cas9 RNP and DNA for gene editing in HMC3

Electroporation was performed in the Lonza 4D Nucleofector X-unit using the pulse codes listed in **Table S3**. The electroporation procedure was as described previously with modifications (29). Briefly, 2 × 10^5^ of HMC3 cells per electroporation reaction were collected by Accutase dissociation. The cells were washed once with DPBS and resuspended in 20 μL of P3 nucleofection buffer (Lonza). Plasmid pmaxGFP (encoding the *turboGFP* reporter gene, Lonza) was added at 0.4 μg to screen for the DNA electroporation condition. Cas9 RNP (targeting the *CD40* gene) was added at 40 pmol to screen for the RNP electroporation condition. The electroporation mixture was gently homogenized by pipetting and then transferred into 16-well strip-format nucleofection cuvettes. Immediately after electroporation, 100 μL of pre-warmed culture medium was added into each nucleofection well to recover the cells in the 37°C incubator for 15 min. After recovery, the cells were transferred to a 6-well culture plate containing 1.4 mL of medium per well, and maintained by the standard culture method. The *turboGFP* expression and *CD40* knockout efficiencies were analyzed by flow cytometry at 24 and 72 hours after electroporation, respectively. Pulse code CA137 was the most balanced condition for Cas9 RNP and DNA electroporation, and was used for all the subsequent gene-editing experiments. For double-cut or double gene knockout experiments, two Cas9 RNP complexes of different sgRNA specificities were co-electroporated (40 pmol + 40 pmol). For gene knock-in, 2 μL of 1 μg/μL of HDR template was co-electroporated with Cas9 RNP. Expression of the modified genes (HA-tagged, GFP-fused and SFFV promoter-inserted) was analyzed by flow cytometry at 72 hours after electroporation.

### Electroporation of Cas9 RNP for gene KO in BV-2

Electroporation was performed using the protocol for HMC3 and a set of BV-2-specific pulse codes listed in **Table S3**. Briefly, 2 × 10^5^ of BV-2 cells per electroporation reaction were collected by Accutase dissociation, washed once with DPBS, and resuspended in 20 μL of SF or P3 nucleofection buffer (Lonza). Cas9 RNP (in complex with sgRNA17 targeting the mouse *CD40* gene) was added at 40 pmol to test the RNP electroporation condition. CM158 + SF is the condition suggested by Lonza and CA137 + P3 is our best condition for HMC3. The other conditions were recommended by Lonza’s optimization scheme. The *CD40* KO efficiencies and cell viability was analyzed by flow cytometry at 72 hours after electroporation. The % CD40-negative cells was determined by staining of mouse CD40. The % viability was determined by Precision beads assay described below and normalized to the unedited cells.

### Gene-editing analysis by Sanger sequencing and ICE

HMC3 cells were harvested by centrifugation at 300 × *g* for 5 min and washed with DPBS once. Cell pellets were lysed by QuickExtraction solution (Lucigen) at 65°C for 15 min, 98°C for 5 min and 4°C for 10 min to extract the genomic DNA. One hundred ng of genomic DNA was used for PCR amplification of the target loci using KAPA HiFi HotStart PCR kit and the primer sets in **Table S4**. After validated by DNA gel electrophoresis, the PCR product was purified by QIAquick PCR Purification Kit, eluted in H_2_O and subjected to Sanger sequencing at the Institute of Biomedical Sciences, Academia Sinica. The % indel was determined on the Synthego website by Inference of CRISPR Edits tool (ICE) using the default setting (https://www.synthego.com/products/bioinformatics/crispr-analysis).

### Analysis of on- and off-target editing by next generation sequencing

Off-target sites were predicted by the CRISPR Design tool on the Benchling website (www.benchling.com) using the published algorism (30). The on-target and off-target genomic sequences were PCR-amplified using KAPA HiFi HotStart DNA Polymerase kit from 300 ng of genomic DNA extracted from the parental or the edited cells by QuickExtraction solution. The primer sequences and thermocycler settings are listed in **Table S4**. The PCR amplicons were purified by gel extraction using QIAQuick Gel Extraction kit (Qiagen). DNA quality was assessed using Qubit DNA quantification (Thermo Fisher Scientific) and size profiling using Fragment Analyzer (Agilent). Nextera XT Index Kit v2 (Illumina) was used to add the dual-barcoded adaptors. The indexing PCR was performed with 5 μl of the amplicon template in 50 μl reactions, and amplified for 8 cycles using 2X KAPA HiFi ReadyMix (KAPA Biosystems). The PCR DNA was purified by AMPure beads (Beckman Coulter), and analyzed by Qubit and Fragment Analyzer. The molar concentration was normalized by quantitative PCR using KAPA Illumina Library Quantification Kit (KAPA Biosystems) prior to library pooling. Next generation sequencing (NGS) of PE2*151 bp was carried out on a MiSeq v2 sequencer using a MiSeq Micro 300 cycles v2 kit (Illumina). From the PE2*150 sequencing run, a total of 13.47 million pass-filter clusters was obtained for the 32 amplicon libraries, averaging 108.5M clusters per sample, with PF rate of 95.68% and >Q30 bases at 98.1% and 95.3% for Read1 and Read2, respectively. The dataset was generated and demultiplexed with BclToFastq 2.1.8 pipeline (Illumina). FASTQ reads were processed by Trimmomatic to trim off the low-quality bases at the 5’ and 3’ ends, and then analyzed by CRISPresso2 (https://crispresso.pinellolab.partners.org/submission) against human reference genome GRCh38 with default parameters. NGS data are available at the NCBI Sequence Read Archive (PRJNA846558). The % Indel of on-target and off-target sites were calculated by the following equation:


% Indel = Number of insertion reads+number of deletion readsTotal number of reads x 100

### Flow cytometry analysis and cell sorting

Flow cytometry was performed on CytoFLEX (Beckman Coulter) at the Flow cytometry core facility in the Institute of Biological Chemistry, Academia Sinica. HMC3 and BV-2 cells were harvested by centrifugation at 500 × *g* for 5 min and washed with 1 mL of ice-cold Flow buffer (DPBS supplemented with 2% of FBS, 25 mM of HEPES, and 0.5 mM of EDTA) once. For surface protein detection, the cells were stained with antibody solution at the manufacturer’s recommended concentration in the dark on ice for 15 min. After staining, the cells were washed with 1 mL of Flow buffer, pelleted at 500 × *g* for 5 min, resuspended in 200 μL of Flow buffer, and transferred to a 5-mL Falcon polystyrene flow tube with a cell strainer snap cap (Corning). For intracellular protein detection, the cells were fixed and permeabilized by Cyto-Fast Fix/Perm Buffer Set (BioLegend) as per manufacturer’s instruction, stained with antibodies at room temperature in the dark for 20 min, and processed similarly for flow cytometry. The gene-edited HMC3 cells were isolated by fluorescence-activated cell sorting (FACS) using FACSAria III (BD Biosciences) at the flow cytometry core facility in the Institute of Biomedical Sciences, Academia Sinica. The isolated cells were collected in 96-well plates at ~10000 cells per 200 μL of medium and expanded by the standard culture method. The expanded cells were validated for protein expression by flow cytometry before storing in liquid nitrogen. All data were analyzed using FlowJo (BD Biosciences) and CytExpert (Beckman Coulter) software.

### Precision beads viability assay

Precision beads assay is as described previously with modifications (29). Both the healthy adherent HMC3 and BV-2 cells and the dead suspended cells were carefully collected and combined for viability analysis (**Figure S1A**). Briefly, the culture medium (1.5 mL) containing the suspended cells and debris was transferred to a 15-mL tube. The adherent cells were dissociated with 300 μL of Accutase for 5 min at 37°C, and then 1.2 mL of DMEM/F12 was added to quench the reaction. The dissociated cells (1.5 mL) were combined with the culture medium in the 15-mL tube to a total of 3 mL. Next, Precision cell count beads (BioLegend) were vortexed thoroughly for 1 min. Three hundred μL of the cell suspension, 10 μL of Precision beads and 3 μL of DAPI (Invitrogen) were mixed in a 1.5-mL microfuge tube. The cell mixture was transferred to the 5-mL flow cytometry tube with cell strainer snap cap, and vortexed for 5 sec immediately before analysis on CytoFLEX to avoid sedimentation. One thousand events of Precision beads were counted by PC5 and APC channels and served as an internal standard to normalize the cell density (gated as P1 in **Figure S1A**). DAPI staining and forward and side scattering were used to determine the viable cells (gated as P2 and shown as black circle in **Figure S1B**). Cell viability was calculated as follows:


% viability = Counts of viable cells in the sampleCounts of viable cells in the mock treatment x 100

### Aβ phagocytosis assay

*In vitro* Aβ phagocytosis assay was adopted from Rangaraju et al., 2018 (31) and Pan et al., 2019 (32) with modifications. Fluorescent fAβ_1-42_ conjugated with HiLyteFluor-488 (Anaspec, 100 μg/vial) was first dissolved in 50 μL of 1% NH_4_OH in DPBS. Then 50 μL of DPBS was added the dissolved fAβ_1-42_ to make 1 μg/μL stock solution (equivalent to 205 μM). Non-fluorescent Aβ_1-42_ (GenicBio) was prepared similarly to 410 μM. To aggregate Aβ_1-42_, equal volumes of fluorescent and non-fluorescent Aβ_1-42_ were mixed at molar ratio of 1:2 to a final concentration of 307.5 μM of total fAβ_1-42_ mixture. The mixture was incubated in a 1.5-mL microfuge tube at 37°C with shaking at 100 rpm in the dark for 24 hours. HMC3 cells were seeded at 5 × 10^4^ cells in 400 μL of DMEM/F12 per well in a 24-well plate. After 24 hours of incubation, 100 μL of medium was removed from the top layer of each well. Per phagocytic reaction, 1.95 μL of the aggregated fAβ_1-42_ was mixed with 100 μL of fresh DMEM/F12, and then transferred to the remaining 300 μL of HMC3 cells in the 24-well. The final fAβ_1-42_ concentration was 1.5 μM in a 400 μL reaction. Non-aggregated Aβ_1-42_ was prepared without overnight aggregation, and was added to the cells by the same method. To block phagocytosis, HMC3 cells were pre-treated with 10 μM of Cytochalasin D (CytoD, Sigma Aldrich) for 1 hour before the assay to inhibit actin polymerization. The CytoD-pretreated cells were kept in 10 μM of CytoD throughout the assay. After incubation with fAβ_1-42_ at 37°C for 12 and 24 hours, the cells were collected by trypsin (0.25%, Gibco) dissociation for 5 min at 37°C, and analyzed by flow cytometry using FITC channel to detect the Fluor 488 signal inside HMC3 cells. In all our phagocytosis assays, we analyzed only the live cells by first gating using FSC and SSC. To distinguish non-specifically-bound fAβ_1-42_ from phagocytosed fAβ_1-42_, we set the fluorescent signal of CytoD-treated cells as background to gate for the fAβ_1-42_-positive cells. Because the parental and mutant HMC3 cells reacted slightly differently to CytoD treatment, the gating of fAβ_1-42_-positive cells was adjusted independently for each cell group using the individual CytoD treatment controls as the fAβ_1-42_-negative cells. Cell viability after 24 hours of fAβ treatment was determined by Precision beads assay as described above, and normalized to the untreated parental or mutant cells. The normalized % viability was calculated by the following equation:


Normalized % viability = Number of viable fAβ-treated cellsNumber of viable untreated cells x 100

### Immunofluorescence staining and confocal microscopy

Glass-bottom microscopy dish (3.5-cm from Ibidi) was coated with 1 mL of poly-D-lysine before 1 × 10^5^ HMC3 cells were seeded. After overnight culture, the aggregated fAβ was added to the cells at 1.5 μM final concentration to initiate phagocytosis for 24 hours. Next, the cells were stained with 1 mL of 100 nM LysoTracker Deep Red (ThermoFisher Scientific) in DPBS at 37°C for 1 hour, and washed 3 times with DPBS. The cells were fixed with 500 μL of 4% paraformaldehyde solution (BioLegend) at room temperature for 10 min, and washed 3 times with DPBS. For intracellular staining, the cells were permeabilized in 500 μL of 0.1% Triton X-100 in DPBS at room temperature for 10 min, and washed 3 times with DPBS. The cells were stained with 1 mL of 5 μg/mL DAPI at room temperature for 30 min and washed 3 times with DPBS. Finally, the cell samples were kept in 1 mL of DPBS for imaging using FV3000 confocal microscopy (Olympus) equipped with a 60X/1.42 oil objective lens at the Biology Core Facility at the Institute of Biological Chemistry, Academia Sinica. Images were taken and processed using FLUROVIEW FV31S-SW software (Olympus) at 2048 × 2048 resolution. Zoomed images were prepared using ImageJ (version 1.53).

### Phagocytosis of apoptotic glioblastoma cells

LN-229 cells were modified to express GFP by transducing with a lentiviral vector encoding the *turboGFP* gene. After transduction, GFP-positive cells were isolated by FACS and clonal expanded. To induce apoptosis, 5 μM of Paclitaxel (Sigma) was added to 2 × 10⁵ cells in a 12-well culture plate for 24 hours. The suspended and adherent LN-229 were collected separately and analyzed for the surface phosphatidylserine (PS) by APC Annexin V Apoptosis Detection Kit using the manufacturer’s protocol (BioLegend). Briefly, the suspended cells were collected from the media, and the adherent cells by Accutase dissociation. The cells were pelleted by centrifugation at 500 × *g* for 5 min and washed with 1 mL of ice-cold Flow buffer once. Next, 5 μL of APC annexin V and 10 μL of DAPI were added to stain PS and the nuclei of dead cells, respectively. The cell samples were analyzed by flow cytometry using APC and PB450 channels to detect apoptotic cells. Suspended LN-229 cells were mostly apoptotic (DAPI^+^ Annexin V^+^). For phagocytosis assay, live (untreated) or apoptotic (Paclitaxel-treated) LN-229 cells were washed with PBS once, and 1 × 10⁵ of LN-229 cells were mixed with 1 × 10⁵ HMC3 cells in 500 μL of DMEM/F12 medium in a 24-well plate. The plate was centrifuged at 120 × g for 2 min to increase cell contact. After 24 hours of co-culture, the cell samples were harvested and stained for CD40 to distinguish HMC3 from LN-229. Ten thousand CD40^+^ cells were counted. The background GFP signal was gated using the untreated HMC3 cells. The presence of CD40 and GFP double-positive cell population indicated HMC3 phagocytosis of LN-229.

### Statistical analysis

The electroporation screening was performed in a single experiment (n=1). All other data were collected from multiple independent experiments, as specified in the methods and figure legends, to determine mean values ± standard deviation (SD). Two-tailed Welch’s unequal variances t-test was used to determine statistical significance in all experiments using GraphPad Prism 9.

## Results

### Analysis of HMC3 cell surface markers

We analyzed the expression of 25 cell surface markers on HMC3 that are reported to regulate phagocytosis in microglia and macrophage (**Figure S2**). We first checked the M1 and M2 polarization markers CD40 and CD206, respectively. We detected the expression of CD40, but not CD206, suggesting that HMC3 was in the pro-inflammatory M1 state. However, recent studies indicate that the M1/M2 paradigm may not accurately reflect the signature of microglial and macrophage immune responses (2). Of the myeloid markers, we observed a low level of CD14, but did not detect the expression of CD11b, CD45, CD68 and HLA-DR. These proteins are also the markers of microglial activation, and their absence suggests that HMC3 is in the resting state (33). We then checked four cytokine and chemokine receptors, and detected only P2RY12 but not IL4RA, TGFBRII and CX3CR1. HMC3 also expressed type III Fcγ receptor CD16, but not type I Fcγ receptor CD64 typically found in macrophages.

We analyzed eight inhibitory and four activating receptors (**Figure S2**). HMC3 expressed a high level of SIRPα and low levels of CD22 and CD96, but no detectable CD33, Siglec10, PD1, LILRB2, and Tim-3. These checkpoint receptors send a “don’t eat me” signal to downregulate microglial activation. Genetic deletion or antibody blockade of SIRPα, CD22, CD33 and PD1 enhance the microglial phagocytic activities against Aβ and glioblastoma, making the receptors attractive therapeutic targets for treating AD and brain tumor (17, 34–36). In the category of activating receptors, HMC3 expressed a high level of CD47 and low level of MERTK, but no detectable TREM2 and CD36. These receptors modulate the activation of microglia in different neurological states. CD36, CD47 and α_6_β_1_ integrin form a receptor complex that binds to Aβ, induces tyrosine kinase-based signaling cascades, and activates microglia (37, 38). MERTK recognizes PS on apoptotic cells as a “eat me” signal to facilitate the pruning of inhibitory post-synapses in the normal brain (39). TREM2 is a phagocytic receptor of Aβ and plays a protective role against AD (40, 41). Mutations that impair TREM2 binding to Aβ are associated with an increased risk for AD and other neurodegenerative diseases (2).

Collectively, our analysis reveals a surface marker profile similar to the review by Russo et al., 2018 (7). However, some discrepancies exist between HMC3 and various reports of primary human microglia, most notably the absence or low expression of CD14, CD36, CX3CR1 and TREM2 in HMC3. We wanted to use CRISPR genome editing to modulate the gene expression in HMC3, generate new mutant cell lines, and expand the application of HMC3 to study microglia-associated diseases.

### Optimization of Cas9 RNP and DNA electroporation in HMC3 cells

We set out to establish a robust electroporation condition for HMC3 to deliver CRISPR genome editing components (Cas9 RNP and synthetic HDR templates) for gene KO and KI (**Figure 1A**). We have previously used Lonza 4D Nucleofector system for Cas9 RNP electroporation and achieved highly efficient gene-editing in human T and natural killer cells (23, 42). Lonza Nucleofector has several advantages over the other electroporation systems including a versatile electroporation format, semi-high throughput capacity and non-toxic carbon-based electrode. However, the drawback of Lonza Nucleofector is that the pulse settings are displayed in proprietary codes and must be optimized experimentally for new cell types and delivery cargos.

We first screened 15 pulse codes in Lonza P3 nucleofection buffer, using pmaxGFP reporter plasmid as the cargo and GFP expression as the readout for DNA electroporation efficiency (**Table S3**). Optimizing DNA electroporation was necessary to deliver synthetic DNA templates to facilitate Cas9-mediated HDR. We used flow cytometry to simultaneously detect the GFP-positive cells and measure cell viability by Precision beads assay (**Figures S1A, S1B**). Most of the 15 pulse codes gave >75% GFP-positive cells except EO100 (**Figure 1B**). The cell viability varied across a broad range from 41% to 89%. We then selected the top seven pulse codes to test for Cas9 RNP electroporation (**Table S3**). We programmed Cas9 with a sgRNA targeting the *CD40* gene, and determine the % CD40-negative cells by flow cytometry as the readout for Cas9 RNP electroporation efficiency (**Figure S1C**). CA137 and CM137 were highly effective in disrupting the *CD40* gene while maintaining high cell viability, yielding ~90% CD40-negative cells and ~75% viability (condition 3 and 4 in **Figures 1C, D**). Our screening has identified CA137 as the most balanced code for plasmid and Cas9 RNP electroporation for HMC3, resulting in high levels of transgene expression, gene KO and cell viability.

### Gene KO by Cas9 RNP electroporation is also highly effective in mouse microglia BV-2

We wanted to know whether gene KO by Cas9 RNP electroporation was also effective in other microglial cells. We adopted the Cas9 RNP editing method for BV-2 cells, a well-established mouse microglial cell line. DNA electroporation in BV-2 has been previously reported by Sui et al., 2007, but using the out-of-production Amaxa nucleofection system (43). We performed another screening, using the same approach as for HMC3, to determine the best Cas9 RNP electroporation condition in Lonza 4D system (**Figure S3A**). We achieved highly efficient *CD40* KO (>95%) in BV-2 in all six conditions we tested; however, the cell viability varied considerably (70-99%). Pulse code CM158 with SF buffer, which is a condition suggested by Lonza, was robust for *CD40* KO (99% CD40-negative cells) but was harsh on the cells (70% viability). Pulse code CA137 with P3 buffer, which is the HMC3 condition, was equally effective at KO and also harsh on the cells. The best condition for BV-2 was pulse code CL135 with SF buffer (**Figures S3B, S3C**). The results show that Cas9 RNP electroporation is an effective method for gene KO in BV-2, and high cell viability can be preserved through optimization of the electroporation condition.

### Cas9 RNP electroporation enables rapid screening of sgRNA

Genomic cleavage by Cas9 RNP creates a site-specific DSB that is predominantly repaired by non-homologous end joining (NHEJ) pathway. NHEJ catalyzes error-prone ligation of the broken DNA ends, introduces random DNA insertion or deletion (indel) at the DSB site, and induces frameshift mutations for gene disruption. Robust Cas9 cleavage requires an efficient sgRNA that targets an accessible genomic site. Screening for robust sgRNA can be a laborious process by the plasmid- and viral vector-based methods. By contrast, the Cas9 RNP platform allows more rapid screening of sgRNA. Multiple sgRNA can be synthesized or purchased commercially to assemble multiple Cas9 RNP to systematically scan a target region for maximum editing efficiency. Electroporation instantaneously delivers the active Cas9 RNP complexes into the cells. Based on our experience in various human cell types, Cas9 RNP editing is often completed within 12-16 hours post electroporation. The cells are ready for DNA sequencing and editing analysis within 24 hours after electroporation as opposed to several days by the plasmid- and viral vector-based editing.

**Figure 2:**
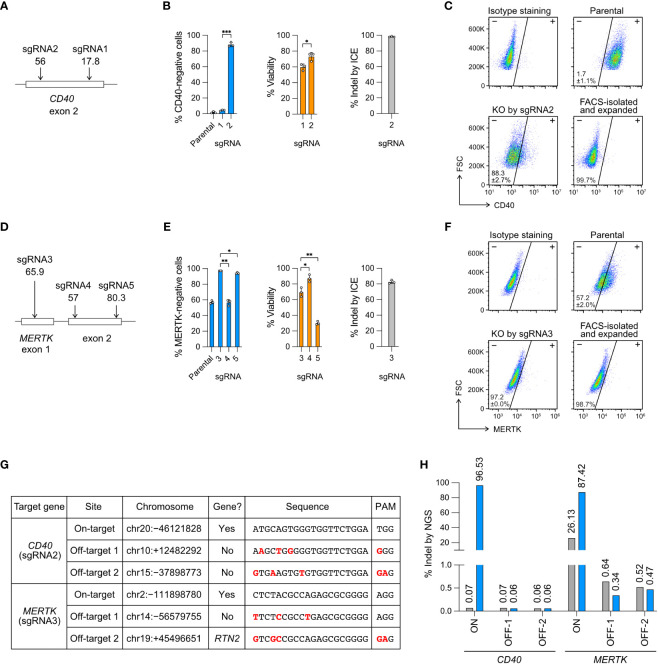
Knockout (KO) of the *CD40* and *MERTK* genes. **(A)** Cas9 was programmed with sgRNA1 and 2 to target the exon 2 of *CD40* gene. *In silico* predicted Cas9 editing efficiencies by CRISPR Design tool are shown below the sgRNA. The higher the score (1-100) indicates the higher predicted efficiency **(B)** The % of CD40-negative cells and viability were measured by flow cytometry, and the % of insertion-deletion (indel) was determined by Sanger DNA sequencing and ICE analysis. **(C)** Representative *CD40* KO flow cytometry plots labeled with the mean % CD40-negative cells and standard deviation (SD). The KO cells were isolated by FACS, expanded for two weeks, and re-analyzed to confirm a stable phenotype. FSC, forward scattering. **(D)** Cas9 was programmed with sgRNA3, 4 and 5 to target the exon 1 and exon 2 of *MERTK*. **(E)** The % of MERTK-negative cells, viability and indel. **(F)** Representative *MERTK* KO flow cytometry plots. The parental HMC3 cells were 57% MERTK-negative. **(G)** Off-target analysis of the *CD40*- and *MERTK*-KO cells by sgRNA2 and 3, respectively. Putative off-target sites were predicted by CRISPR Design tool. Sequence mismatches between the on-target and off-target sites were marked in red. **(H)** The % indel at the *CD40* and *MERTK* on- and off-target sites were determined by NGS and CRISPresso2 analysis. Data are shown as mean ± SD of three independent experiments (n = 3). Two-tailed Welch’s unequal variances t test was used to test for statistical significance. *, P ≤ 0.05; **, P ≤ 0.01; ***, P ≤ 0.001.

To demonstrate the versatility of Cas9 RNP electroporation, we prepared two sgRNA to target the *CD40* gene and three sgRNA to the *MERTK* gene at the early exons using the CRISPR Design tool on Benchling website (**Figures 2A, D**). CRISPR Design tool provides *in silico* prediction of on-target and off-target editing scores for sgRNA selection. We electroporated the individual Cas9 RNP into HMC3, and analyzed the cells three days after electroporation to allow turnover of CD40 and MERTK proteins. We performed flow cytometry to measure the % CD40- or MERTK-negative cells and determine the % viability. We also extracted the genomic DNA from the edited cell population, PCR-amplified the target regions for Sanger sequencing, and determine the indel frequency (% indel) by ICE analysis. Using this workflow, we were able to rapidly validate multiple sgRNA in HMC3, compare the editing efficiencies at protein and DNA levels, and identify the most robust sgRNA for the target gene.

For *CD40* KO, sgRNA2 was more efficient than sgRNA1, yielding 88% CD40-negative cells, 75% viability and 98% indel (**Figure 2B**). For *MERTK* KO, sgRNA3 was more efficient than sgRNA4 and sgRNA5, yielding 97% MERTK-negative cells (from 57% parental cells), 69% viability and 83% indel (**Figure 2E**). We further isolated the KO cells by FACS and expanded the cells for several passages to confirm permanent inactivation of the target genes (**Figures 2C, F**). Our results reveal that the *in silico* prediction of on-target editing efficiency does not always agree with the *in cellulo* editing efficiency, emphasizing the need to validate the sgRNA in the cells (**Figures 2A, D**).

### Cas9 RNP editing is precise with no detectable off-target effect

To ensure the precision of Cas9 RNP editing, we analyzed the FACS-sorted and expanded *CD40*- and *MERTK*-KO cells by NGS to detect Cas9 off-target editing. The potential off-target sites were predicted *in silico* by the CRISPR Design tool using the algorithm reported by Hsu et al. (30). We selected two top predicted off-target sites for each of the *CD40*- and *MERTK*-targeting sgRNA (**Figure 2G**), and performed the amplicon sequencing on an Illumina MiSeq system.

We first checked the on-target editing efficiencies. The NGS results showed 96.5% and 87.4% indel at the *CD40* and *MERTK* target sites, respectively (**Figures 2H**, **S4**, **S5**). The NGS results were comparable to those by Sanger sequencing and ICE analysis at 98.3% and 82.7% indel for the *CD40* and *MERTK* sites, respectively (**Figures 2B, E**). Surprisingly, we detected 26.3% of sequence heterogeneity at the *MERTK* target region in the unedited parental HMC3 cells (**Figures 2H**, **S5**). The polymorphism likely accounted for the MERTK-negative cells (57%) in the parental HMC3, suggesting that the parental HMC3 was a mixed cell population of heterogeneous *MERTK* genotypes (**Figure 2F**). We then checked the four predicted off-target sites in the KO cells. The off-target indel frequencies of the KO cells were all at the baseline levels as compared to the parental HMC3 (**Figure 2H**). Collectively, the NGS results from the *CD40-* and *MERTK-*KO cells show that Cas9 RNP editing is precise when the sgRNA is carefully designed to avoid off-target editing. Further analyses are needed to determine whether the *MERTK* mutations exit in the original cell stock or arise in our cell culture.

### Cas9 RNP electroporation allows multiplexed gene editing

**Figure 3:**
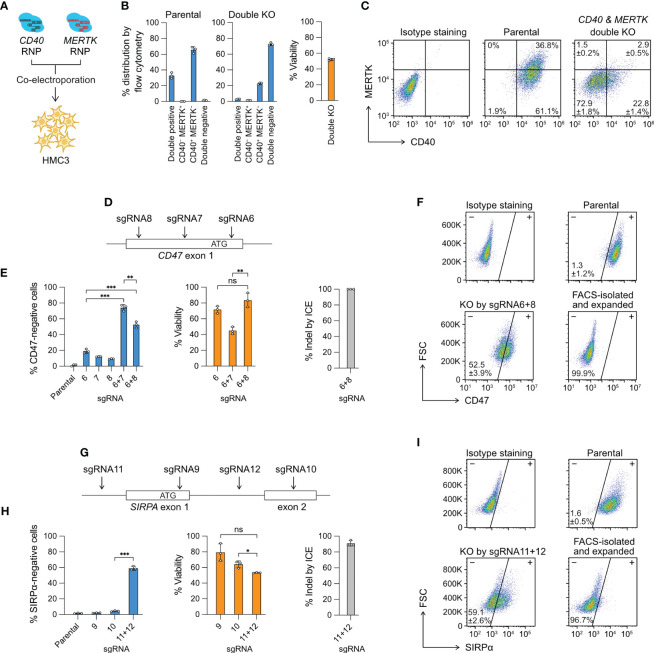
Multiplexed editing in a single electroporation reaction. **(A)** *CD40*- and *MERTK*-targeting Cas9 RNP complexes were co-electroporated into HMC3 for double KO. **(B)** The % distribution of CD40 MERTK double positive, CD40-negative MERTK-positive (CD40^−^ MERTK^+^), CD40-positive MERTK-negative (CD40^+^ MERTK^−^) and CD40 MERTK double negative cells were determined by flow cytometry. The % viability after the double KO experiment is also shown. **(C)** Representative flow cytometry plots of the parental and double KO cells, labeled with the mean % distribution and SD. **(D)** *CD47* KO by the double-cut strategy at the exon 1 region using different combinations of sgRNA6, 7 and 8. **(E)** The % of CD47-negative cells, viability and indel generated by single or combined sgRNA. **(F)** Representative flow cytometry plots of the parental and sgRNA6 + 8 KO cells, labeled with the mean % CD47-negative cells and SD. The KO cells were isolated by FACS, expanded for two weeks, and re-analyzed to confirm a stable phenotype. **(G–I)** The data set for *SIRPA* KO by the same double-cut strategy. Data are shown as mean ± SD of three independent experiments (n = 3). Two-tailed Welch’s unequal variances t test was used to test for statistical significance. *, P ≤ 0.05; **, P ≤ 0.01; ***, P ≤ 0.001; ns, not significant.

Multiple Cas9 RNP complexes of different target specificities can be co-electroporated in a single reaction for multiplexed editing. This approach is technically more challenging with the plasmid- or viral vector-based methods because co-transfection or co-transduction of the *cas9* and multiple sgRNA coding sequences is not efficient. Here we provide two demonstrations of multiplexed editing by Cas9 RNP electroporation. In the first demonstration, we co-electroporated the *CD40* and *MERTK* Cas9 RNP to simultaneously KO two genes (**Figure 3A**). The parental HMC3 consists of mainly two populations: 36.8% CD40^+^ MERTK^+^ and 61.1% CD40^+^ MERTK^−^ (**Figures 3B, C**). After the double KO, the cell population shifted to mainly 22.8% CD40^+^ MERTK^−^ and 72.9% CD40^−^ MERTK^−^. The cell viability reduced to ~50% due to the increased Cas9 RNP dosage. To ensure the *CD40*- and *MERTK*-targeting sgRNAs did not have non-specific cross-reactivities, we analyzed the MERTK level in the *CD40* KO cells and CD40 level in the *MERTK* KO cells. We did not detect any non-specific fluctuation in the protein levels (**Figure S6**). The result shows that double KO by the co-electroporation of two Cas9 RNP complexes is specific and efficient as single KO.

In the second demonstration, we electroporated two Cas9 RNP complexes to “double cut” at the target gene to create a gene lesion and enhance KO efficiency. We designed three sgRNA to target the *CD47* exon 1 region; however, the individual sgRNA was inefficient at disrupting the *CD47* gene at rates of 10-20% (**Figures 3D, E**). We then co-electroporated the Cas9 RNP complexes consisting of sgRNA6 + 7 and sgRNA6 + 8, and observed markedly enhanced *CD47* disruption in both combinations at 50-70% (**Figures 3E, F**). Similarly, we used the same double-cut strategy to KO the *SIRPA* gene, where we failed to induce disruptive indels by sgRNA9 and sgRNA10, respectively (**Figure 3G**). We then co-electroporated sgRNA11- and sgRNA12-Cas9 RNP to delete the exon 1 and achieved ~60% KO efficiency (**Figures 3H, I**). The double-cut strategy in the *CD47* and *SIRPA* KO deletes a critical region of the genes, which contains the ribosome binding site (Kozak sequence) and the start codon sequence, both essential for protein synthesis. In comparison, the single-cutting approach has a 2/3 probability of generating a frameshift mutation when the indel from NHEJ repair is not in the factor of three nucleotides. Together, our results demonstrate that for hard-to-edit genes, double-cut strategy is more effective than single-cut at inducing more disruptive gene lesion.

### Co-electroporation of HDR templates enables targeted gene insertion

**Figure 4:**
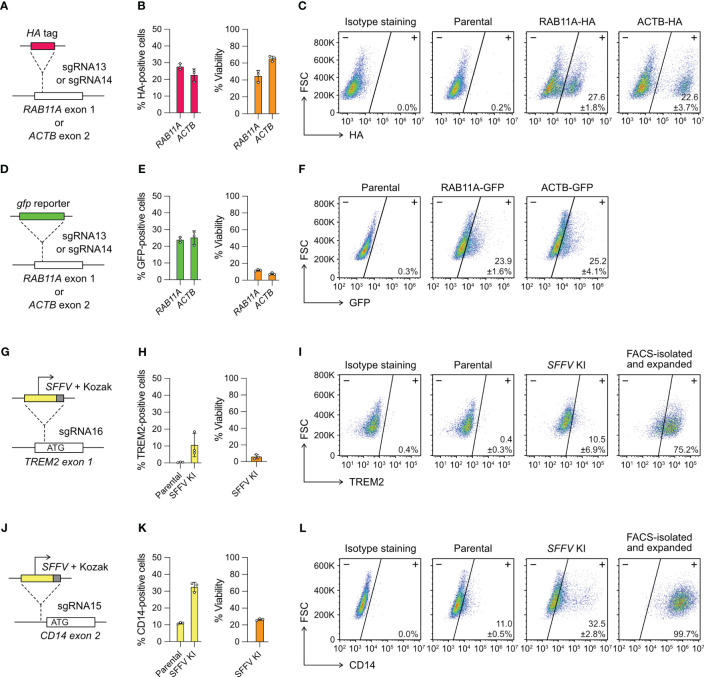
Targeted gene insertion by Cas9 RNP-mediated knock-in (KI). **(A)** Insertion of a HA tag-coding sequence at the *RAB11A* and *ACTB* gene as specified by sgRNA13 and 14, respectively. **(B)** The % HA-positive cells and viability as determined by flow cytometry. **(C)** Representative flow cytometry plots labeled with the mean % HA-positive cells and SD. **(D–F)** The design and results of *gfp* KI at the *RAB11A* and *ACTB* gene. GFP-positive cells were detected directly by GFP fluorescence without antibody staining, and hence no isotype staining was needed. **(G–I)** The design and results of SFFV promoter KI to activate TREM2 expression. **(J–L)** The design and results of SFFV promoter KI to activate CD14 expression. The TREM2- and CD14-positive cells were isolated by FACS, expanded for two weeks, and re-analyzed to confirm stable phenotypes. Data are shown as mean ± SD of three independent experiments (n = 3). Detailed illustrations and complete DNA sequences of the HDR templates are in **Figure S7** and **Table S2**.

Cas9-mediated HDR allows precise gene modifications and insertion of novel DNA sequences such as epitope tags and reporter genes. The process requires the co-electroporation of a synthetic DNA repair template for Cas9-mediated HDR. However, HDR is a tightly regulated process at specific cell cycle stages and is often less efficient than NHEJ. Furthermore, Cas9-mediated HDR is confined by the cellular toxicity of synthetic DNA, which results in poor survivability of the edited cells. To assess whether Cas9-mediated HDR was feasible in HMC3, we programmed Cas9 to target the exons of *RAB11A* and *ACTB* housekeeping genes to insert a *HA* tag and a *gfp* reporter gene (**Figures 4A, D**). The expression of HA tag and GFP was possible only when the coding sequences were integrated precisely in-frame with the *RAB11A* and *ACTB* transcripts.

We designed single-strand DNA (ssDNA) templates for the *HA*-tag KI, but double-strand DNA (dsDNA) for *gfp* KI because of large insert size (**Figure S7**). We used flow cytometry to detect the HA- and GFP-positive cells three days after editing. The % HA-positive cells were at 20-30% in the *RAB11A* and *ACTB* KI experiments (**Figures 4B, C**). The viabilities were 40-60%, similar to the range we observed in the KO experiments. The % GFP-positive cells were also at 20-30%, indicating that the KI efficiencies of *HA* tag and *gfp* gene were similar (**Figures 4E, F**). However, the dsDNA templates were toxic to HMC3 and the viabilities of *gfp* KI experiments reduced to ~10% at both loci. The results show that Cas9-mediated HDR enables precise insertion of exogenous DNA sequences into HMC3 genome, but the toxicity of dsDNA templates reduces the viability of the edited cells.

### Promoter insertion activates the silenced endogenous genes

In addition to epitope tags and fluorescent reporter genes, Cas9-mediated HDR can potentially insert a synthetic promoter to activate the silenced endogenous genes and restore the gene functions. To test this idea, we targeted the *TREM2* and *CD14* genes, both are not expressed in the parental HMC3. TREM2 and CD14 are implicated in microglial response to Aβ and the progression of AD. However, the lack of TREM2 and CD14 renders HMC3 unsuitable to study the receptor functions. To overcome this limitation, we programmed Cas9 to introduce a DSB between the endogenous promoter and the start codon to insert a SFFV promoter sequence by HDR. Transcription from the SFFV promoter can bypass the silenced endogenous promoter and hence activate the TREM2 and CD14 expression.

We designed two different HDR templates for the *TREM2* and *CD14* loci, each encoding the SFFV promoter, Kozak sequence and gene-specific homology arms (**Figures 4G, J**, **S7**). Three days after the co-electroporation of Cas9 RNP and HDR templates, the TREM2 and CD14 expression were analyzed by flow cytometry. The SFFV-KI at the *TREM2* site was inefficient, resulting in ~10% TREM-positive cells at <10% viability (**Figure 4H**). The KI efficiency at the *CD14* site was higher, yielding ~30% CD14-positive cells at ~20% viability (**Figure 4K**). In both KI experiments, the edited cells appeared stressed by the electroporation of dsDNA templates as seen in the case of *gfp* KI. The results also show that the KI efficiency is target dependent. We isolated the TREM2- and CD14-positive cells by FACS. Despite the initial toxicity, the edited cells recovered and maintained stable TREM and CD14 expression after several passages (**Figures 4I, L**). Using the promoter KI strategy, we successfully activated the endogenous *TREM2* and *CD14* genes, and generated stable TREM2- and CD14-expressing HMC3 cell lines.

### CRISPR editing enhances Aβ phagocytosis in HMC3 cells

Microglia share a common lineage with monocyte-derived macrophages and are highly capable of phagocytosis to remove damaged cells and misfolded proteins (5). We were interested in the microglial phagocytosis of Aβ aggregates, and wanted to know whether the activity could be enhanced by genome editing. We focused on the surface receptors CD14, CD47 and TREM2 implicated in Aβ phagocytosis. CD14 and CD47 are reported to bind to Aβ and stimulated microglial activation in mouse models (44, 45), although the activation role of CD47 is still debated (46). TREM2 facilitates the uptake of Aβ and is a critical factor of AD progression (41). Loss of function *TREM2* mutations (for examples R47H and R62H) increases the risk of AD (47). We sought to generate HMC3 mutant cells to validate the roles of these receptors.

**Figure 5:**
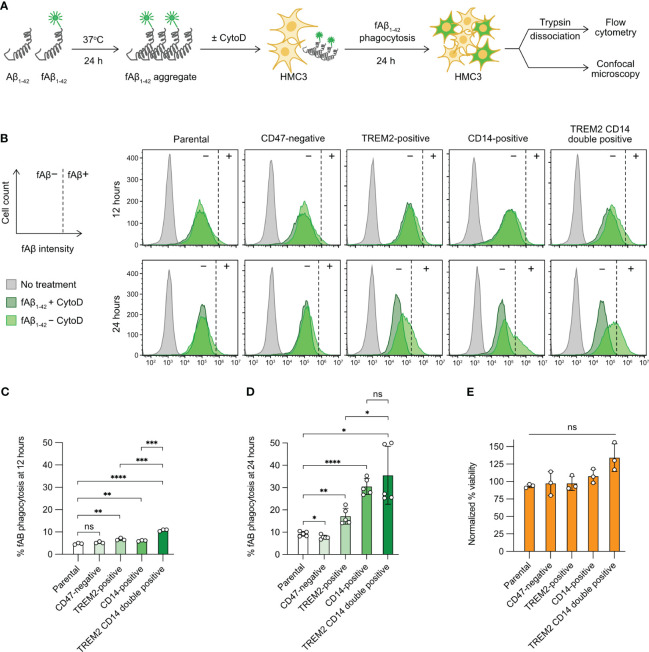
Aβ phagocytosis of the gene-edited HMC3 cells. **(A)** Workflow of the *in vitro* Aβ phagocytosis assay using the aggregated and fluorescently label Aβ synthetic peptides (fAβ_1-42_). As a negative control, HMC3 cells were treated with cytochalasin D (CytoD) to inhibit actin polymerization and hence block phagocytic activity. The fluorescent signal (HiLyteFluor-488) of the engulfed fAβ_1-42_ was measured by flow cytometry and visualized by confocal microscopy. **(B)** Representative flow cytometry plots of fAβ_1-42_ phagocytosis of the parental, CD47-negative, TREM2-positive, CD14-positive and TREM2 CD14 double-positive cells at 12 and 24 hours of the assay. The fluorescent signal of the CytoD-treated cells was regarded as background (marked as −) and was used for the gating of fAβ_1-42_-positive cells (marked as +). The gating for fAβ_1-42_-positive cells was adjusted independently based on the individual CytoD treatment controls to compensate for small variations in CytoD response. The percentages of fAβ_1-42_ phagocytosis at 12 **(C)** and 24 hours **(D)**. Data are shown as mean ± SD of three (n = 3) and five (n = 5) independent experiments for 12 and 24 hours, respectively. **(E)** % viability of the fAβ-treated cells at 24 hour of the assay as normalized to the untreated cells. Data are shown as mean ± SD of three independent experiments (n = 3). Two-tailed Welch’s unequal variances t test was used to test for statistical significance. ns, no significant; *, P ≤ 0.1; **, P ≤ 0.01; ***, P ≤ 0.001; ****, P ≤ 0.0001.

HMC3 expresses CD47, but lacks TREM2 and CD14 (**Figure S2**). We overcame this limitation by Cas9-mediated KO and KI to generate stable CD47-negative, TREM2-positive and CD14-positive mutants as described above (**Figures 3F**, **4I, L**). We adopted an *in vitro* assay from Rangaraju et al., 2018 (31) and Pan et al. (32) to assess the phagocytic activity of the individual mutants using fAβ_1-42_ as a substrate (**Figure 5A**). The fAβ_1-42_ phagocytosis was quantitated by flow cytometry based on the fAβ_1-42_ fluorescent signal. The assay was optimized to avoid false signals from non-specific adhesion of fAβ_1-42_ to cell surface (**Figure S8A**). First, we treated the HMC3 cells with trypsin prior to flow cytometry analysis to remove surface bound fAβ_1-42_. Second, we disrupted fAβ_1-42_ phagocytosis by CytoD, which is a cell-permeable inhibitor of actin polymerization, in a dosage dependent manner (**Figures S8B, S8C**). Finally, we observed higher levels of phagocytosis towards the aggregated fAβ_1-42_ than the non-aggregated form (**Figure S8B**). Taken together, our setup shows that HMC3 cells respond specifically to the aggregated form of Aβ, and Aβ phagocytosis is an active process dependent on actin polymerization.

Next, we compared the parental HMC3 with the three mutants. The gating of fAβ_1-42_-positive cells was adjusted slightly and independently for each cell group using the individual CytoD treatment controls as the fAβ_1-42_-negative cells (dark-green peaks, **Figure 5B**). The adjustment was to compensate for small variations in CytoD response in individual cell groups at different assay time points. We measured the percentages of fAβ_1-42_-positive cells at 12 and 24 hours after fAβ_1-42_ addition. At 12 hours, the levels of fAβ phagocytosis were low at 5-10% in all cell groups (**Figure 5C**). At 24 hours, the parental HMC3 and CD47-negative cells had comparable fAβ_1-42_ phagocytosis at ~10% (**Figure 5D**). The TREM2- and CD14-positive mutants increased fAβ_1-42_ phagocytosis to 15% and 30%, respectively (**Figure 5D**). We also constructed a TREM2 and CD14 double-positive mutant (**Figures S9A, S9B**). However, the phagocytosis of double-positive mutant did not increase significantly over the CD14-positive cells (**Figure 5D**). Regardless of phagocytic activities, fAβ_1-42_ treatment did not affect the viability of parental and mutant cells (**Figures 5E**, **S10**). Collectively, our results show that the CRISPR activation of TREM2 and CD14 increases the Aβ phagocytosis of HMC3, confirming that the receptors are important for efficient microglial clearance of Aβ aggregates.

### Confocal microscopy shows co-localization of the phagocytosed Aβ with lysosome

**Figure 6:**
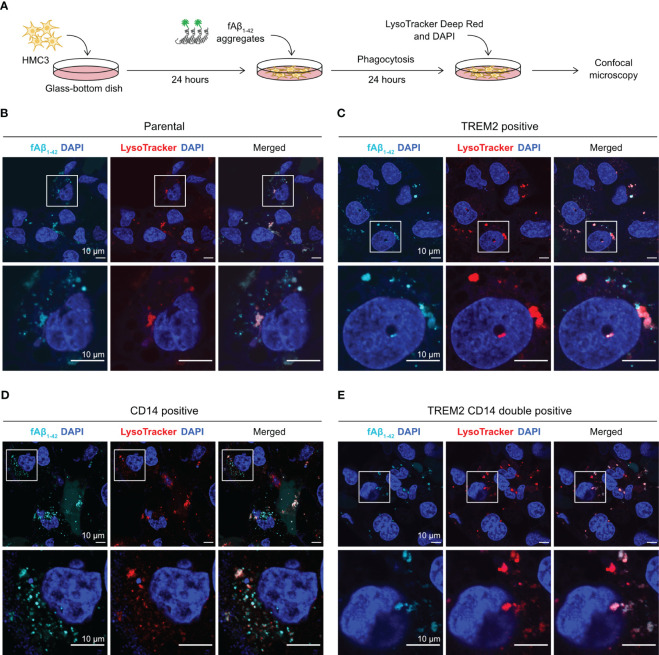
Visualization of fAβ_1-42_ phagocytosis by fluorescent confocal microscopy. **(A)** HMC3 cells were seeded in the glass-bottom microscopy dish and incubated with fAβ_1-42_ aggregates for 24 hours. LysoTracker Deep Red and DAPI were added to detect lysosome formation and nuclei, respectively. **(B)** Images of the parental HMC3. The signal of fAβ_1-42_ aggregates is shown in cyan, LysoTracker in red and DAPI in blue. Images were enlarged (marked by white boxes) to better visualize the puncta and distribution of fAβ_1-42_ aggregates and lysosomes. Images of TREM2-positive cells **(C)**, CD14-positive cells **(D)** and TREM2 CD14 double positive cells **(E)** are displayed in the same layout. The scale bars are in 10 μm.

We used fluorescent confocal microscopy to confirm the internalization of phagocytosed Aβ in HMC3 cells. We repeated the fAβ_1-42_ phagocytosis assay on a glass-bottom microscopy dish, and stained the cells with LysoTracker Deep Red to detect lysosome formation (**Figure 6A**). LysoTracker is a cell membrane-permeable dye that stains specifically to acidic organelles and is used to visualize the activated lysosomes. Using this setup, we analyzed the fluorescent signal and cellular distribution of phagocytosed fAβ_1-42_. In the parental and mutant cells, we observed multiple puncta of fAβ_1-42_ inside the cells (**Figures 6B–E**). The fAβ_1-42_ puncta varied in size, shape and number between the cells. The majority of fAβ_1-42_ puncta, but not all, co-localized with the LysoTracker signal, indicating that the phagocytosed fAβ_1-42_ were sequestered inside the lysosomes. Although difficult for direct fAβ_1-42_ quantitation, the microscopic images provide an important visual confirmation of the fluorescent signal of phagocytosed fAβ_1-42_.

### HMC3 cells phagocytose apoptotic glioblastoma cells

**Figure 7:**
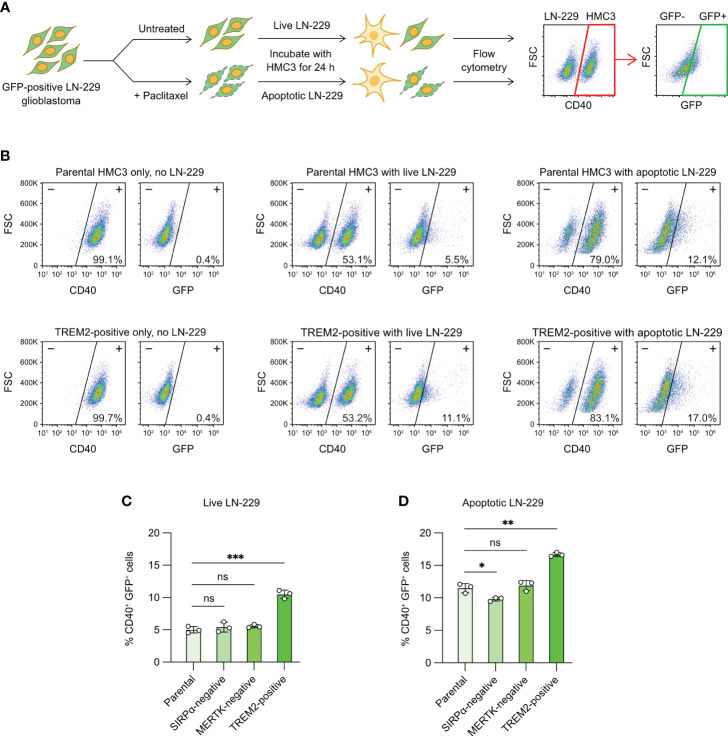
Microglial phagocytosis of apoptotic glioblastoma cells. **(A)** Workflow of fluorescent tumor phagocytosis assay. GFP-expressing LN-229 glioblastoma cells were treated with Paclitaxel to induce apoptosis. Live or apoptotic LN-229 cells were co-cultured with the parental or mutant HMC3 cells for 24 hours. The mixed cell population was then analyzed by flow cytometry. HMC3 phagocytosis of LN-229 resulted in the appearance of CD40^+^ (HMC3 marker) and GFP^+^ (engulfed LN-229) cell population as detected by flow cytometry. The percentage of CD40^+^ GFP^+^ cells served as a measure for tumor phagocytosis. **(B)** Representative flow cytometry plots showing the co-culture of the parental HMC3 and TREM2-positive mutant with live or apoptotic LN-229. **(C)** % CD40^+^ GFP^+^ cells after the co-culture with live LN-229. **(D)** % CD40^+^ GFP^+^ cells after co-culture with apoptotic LN-229. Data are shown as mean ± SD of three independent experiments (n = 3). Two-tailed Welch’s unequal variances t test was used to test for statistical significance. ns, no significant; *, P ≤ 0.1; **, P ≤ 0.01; ***, P ≤ 0.001.

Tumor-associated microglia play crucial roles in the development of glioblastoma (5). We wanted to know whether CRISPR editing could modify the phagocytic characteristic of HMC3 towards human glioblastoma cells. We were interested in SIRPα, MERTK and TREM2 because of their roles in the phagocytosis of cancer and apoptotic cells. SIRPα is an inhibitory receptor that binds to CD47 on cancer cells and suppresses microglial and macrophage phagocytosis (48). MERTK is an activating receptor that recognizes the exposure of PS on the surface of apoptotic cells (39). TREM2 is shown to facilitate the clearance of cancer and damaged neuronal cells by macrophages and microglia (49, 50). To test our CRISPR-edited cells, we set up a fluorescent phagocytosis assay to target a modified LN-229 cell line, which constitutively expressed GFP (**Figure 7A**). We co-cultured HMC3 with GFP^+^ LN-229 for 24 hours, and analyzed the phagocytic activity by flow cytometry to detect the transfer of GFP signal to CD40^+^ HMC3 cells. The presence of CD40^+^ GFP^+^ cells indicated the phagocytosis of LN-229 by HMC3.

The co-culture of live LN-229 with parental HMC3, SIRPα-negative and MERTK-negative cells yielded low % GFP^+^ CD40^+^ cells (~5%), indicating low activities of tumor phagocytosis (**Figures 7B, C**, **S12**). By contrast, TREM2-positive mutant showed two-fold increase in phagocytosis at ~10%. We repeated the assay with apoptotic LN-229. We induced apoptosis of LN-229 by Paclitaxel treatment (**Figure S11A**). After 24 hours of drug treatment, the adherent LN-229 cells dissociated from the culture plate and became suspended. Annexin V assay revealed that the suspended LN-229 cells were mostly apoptotic as compared to the adherent cells (**Figures S11A, S11B**). The co-culture of apoptotic LN-229 increased the CD40^+^ GFP^+^ cell population to ~10% in the parental, SIRPα-negative and MERTK-negative cells, showing that microglial phagocytosis was more efficient on apoptotic glioblastoma cells (**Figures 7B, C**, **S12**). TREM2-positive mutant again yielded more CD40^+^ GFP^+^ cells at ~17%. Collectively, our results demonstrate that activation of the *TREM2* gene enhances the HMC3 phagocytosis of LN-229 in both live and apoptotic forms (**Figure 7D**). The ablation of SIRPα and MERTK receptors in HMC3 did not change the phagocytosis of LN-229, but more work is needed to ensure the signaling pathways of SIRPα and MERTK receptors are functional in HMC3.

## Discussion

HMC3 is a highly valuable tool to study human microglial functions and validate the findings from animal models. To further expand its research applications, we have established a robust CRISPR genome editing platform, by the electroporation of Cas9 RNP and synthetic DNA templates, to enable precise gene KO and KI in HMC3. Our platform is also applicable and highly effective for gene KO in BV-2, a well-established mouse microglial cell line. For proof-of-concept demonstrations, we modified the genes implicated in Aβ and glioblastoma phagocytosis, and showed that the phagocytic activities could be altered by manipulating the expression of crucial activating and inhibitory receptors.

Genome editing by Cas9 RNP electroporation is a popular approach for human immune cell that are difficult to genetically modify by the standard plasmid- and viral vector-based methods. Cas9 RNP has several advantages. First, recombinant Cas9 protein and synthetic sgRNA can be prepared in advance, stably stored in the freezer, and pre-assembled *in vitro* into active RNP complexes for electroporation on-demand. Second, gene editing by the pre-assembled Cas9 RNP is rapid and transient because Cas9 protein has a short half-live (~18 hours) in the cells and is quickly degraded to avoid undesirable editing activities. Third, there is no foreign genetic material in the form of *cas9* and sgRNA-encoding plasmids or viral vectors. The absence of plasmid and viral vector reduces the risk of off-target editing associated with prolonged Cas9 and sgRNA expression and residual editing activity. Rapid and safe gene editing is particularly important to primary immune cells whose *ex vivo* life span is limited. It would be interesting to explore Cas9 RNP editing in the primary human microglia.

Promoter KI is a precise and effective method to restore the expression of endogenous genes as opposed to the conventional plasmid- and lentiviral-based transgene expression. Re-activating genes at their endogenous loci affords more stable gene expression than plasmid transfection, and is more site-specific than random gene integration by lentiviral transduction. Although promoter KI in HMC3 is confined by the toxicity of synthetic DNA repair templates, the edited cells recovered well from the initial stress. Cell isolation by FACS helps overcome the limitations of DNA toxicity and HDR efficiency to obtain stable and homogenous population of the edited cells. The promoter KI strategy can also be tweaked to incorporate different promoter sequences to tune gene expression level, although some promoters have longer coding sequences and may render the KI less efficient. The caveat of a successful promoter KI experiment is that the HDR needs to be of high efficiency to generate sufficient numbers of the edited cells, or the small population of edited cells must be expanded to sufficient numbers over an extended period of cell culture. We anticipate that the promoter KI approach would be more challenging in primary microglia, which do not yet have a robust *ex vivo* expansion condition.

Genome editing opens the possibility to investigate the mechanism and regulation of microglial phagocytosis using HMC3 cells, as an alternative to mouse and primary human microglia. Our CRISPR-edited TREM2- and CD14-positive mutants are capable of efficient Aβ phagocytosis, although the double-positive mutant did not increase the activity further, potentially due to saturation of the downstream activation signal. It would be fascinating to understand the activities and regulations of TREM2 and CD14 in primary microglia in a healthy CNS and AD patients. The physiological levels of TREM2 and CD14 are likely different to the SFFV-driven expression in the gene-edited HMC3. Several questions remain unanswered. Are TREM2 and CD14 upregulated in microglia to meet the higher demand of Aβ clearance? Do TREM2 and CD14 function independently or cooperatively to improve the scavenging of Aβ aggregates? Do microglia have enough capacity to process and degrade the excess influx of Aβ aggregates? The answers could help develop TREM2- and CD14-targeted therapy to treat AD and related neurodegenerative diseases.

In our study of glioblastoma phagocytosis, we observed that the apoptosis of cancer cells was a critical signal to activate HMC3 phagocytosis. Microglia is known to remove damaged and apoptotic neuronal cells to maintain CNS homeostasis, but the role of microglial phagocytosis of apoptotic glioblastoma is less understood. In our *in vitro* assay, we observed an increase in HMC3 phagocytosis of both live and apoptotic LN-229 cells by activating TREM2 expression. However, the disruption of *SIRPA* and *MERTK* genes had minimal effect on the phagocytosis of LN-229. Since glioblastoma phagocytosis is a more complex than Aβ phagocytosis, the process likely involves other protein factors than the single receptors. Future work is needed to validate the downstream signaling of the receptors and also to identify other protein factors involved in the process.

In summary, CRISPR genome editing by Cas9 RNP electroporation is a powerful technique to engineer human microglial HMC3 cells. Using our editing platform, we can genetically modify HMC3 cells with high versatility, efficiency and precision. Systematic gene-editing can help determine surface receptors that are important to microglial phagocytosis, an activity that is crucial to the development of CNS and the prevention of neurological disorders. Furthermore, the gene-edited HMC3 cells can potentially be used to screen for drug targets and develop therapeutic strategies to treat brain diseases.

## Data availability statement

The datasets presented in this study can be found in online repositories. The names of the repository/repositories and accession number(s) can be found below: https://www.ncbi.nlm.nih.gov/, PRJNA846558.

## Author contributions

JC-YC, C-YW, and SL conceived and designed this study. JC-YC and C-YW performed all the experiments. SL supervised the study and wrote the manuscript. All authors contributed to the article and approved the submitted version.

## Supplementary material

Supplementary Figure 1 Optimization of Cas9 RNP electroporation. **(A)** Workflow of Precision beads viability assay. Adherent cells, suspended cells and cell debris were all collected and combined in a tube for cell counting by flow cytometry. Precision beads and DAPI dye were then added to the cell suspension. Cell density was determined per 1000 counts of Precision beads (gated as P1). **(B)** Flow cytometry plots from the Precision beads assay. HMC3 cells were electroporated with Cas9 RNP by five different pulse codes. In the mock treatment, the cells were processed by the same procedure and resuspended in the electroporation buffer, but were not electroporated. The viable HMC3 cells were gated by FSC and side scattering (SSC) as marked by P2 (black circle). Dead cells were DAPI-positive (red). Viable cell counts (%) were normalized to the mock treatment. **(C)** *CD40* KO efficiencies of the five different pulse codes were determined by cell surface staining with anti-CD40 antibodies.

Supplementary Figure 2 Expression profile of surface markers of the parental HMC3 cells. The receptors are categorized by their functions: polarization markers, cytokine and chemokine receptors, myeloid markers, Fc receptors, inhibitory receptors and activating receptors. Isotype control staining is shown in gray peaks, and the marker-specific staining is in colored peaks. The y- and x-axis are cell count and expression level, respectively.

Supplementary Figure 3 Screening of Cas9 RNP electroporation conditions for *CD40* KO in BV-2 mouse microglia. **(A)** Screening was performed using a set of BV-2-specific conditions (color-coded). The % CD40-negative cells and % normalized viability were determined at 72 hours after electroporation. **(B)** Flow cytometry plots showing the CD40 KO efficiencies of different electroporation conditions. The CD40-negative cells were gated by isotype staining. **(C)** Flow cytometry plots showing absolute cell counts from Precision beads assay and the % normalized viability under different electroporation conditions. Viable cells were marked by black cycles.

Supplementary Figure 4 Analysis of on- and off-target editing by *CD40*-targeting Cas9 RNP. The NGS raw data were analyzed and the results were generated by CRISPresso2. The binding positions of sgRNA2 are marked by gray lines. The predicted Cas9 cleavage positions are marked by arrows and dot lines. Red boxes represent base insertions, whereas hyphens represent deletions. Base substitutions are marked by bold letters.

Supplementary Figure 5 Analysis of on- and off-target editing by *MERTK*-targeting Cas9 RNP. The NGS raw data were analyzed and the results were generated by CRISPresso2. The binding positions of sgRNA3 are marked by gray lines. Red boxes represent base insertions, whereas hyphens represent deletions. Base substitutions are marked by bold letters. The predicted Cas9 cleavage positions are marked by arrows and dot lines. The parental cell population contained about 30% mutations at the *MERTK* target region.

Supplementary Figure 6 Control staining of CD40 and MERTK in the *MERTK*- and *CD40*-KO cells. Representative flow cytometry plots showing that the KO of *CD40* and *MERTK* genes do not change the MERTK and CD40 expression, respectively.

Supplementary Figure 7 Cas9-mediated homology-directed repair (HDR) for the insertion of HA epitope tag, *gfp* reporter gene and SFFV promoter. **(A)** The design of HA-tag and *gfp* templates for insertion at the *RAB11A* exon 1 region. The HA-tag template is a 200-nt single-stranded synthetic DNA (ssDNA) and the *gfp* template is a 1350-nt double-stranded DNA (dsDNA) generated by PCR. The sgRNA13 binding site is labeled in blue and the PAM sequence in red. The predicted Cas9 cleavage site is marked by a red arrow. The *gfp* gene is flanked by glycine and serine (GS) linkers. **(B)** The design of HA-tag and *gfp* templates for insertion at the *ACTB* exon 2 region by sgRNA14. **(C)** The design of SFFV template for insertion at the *TREM2* exon 1 region by sgRNA16. The SFFV template encodes the SFFV promoter and Kozak sequences, flanked by homology arms. Successful HDR will replace the region between sgRNA16 cut site and the start codon with the SFFV and Kozak sequences. **(D)** The design of SFFV template for insertion at upstream of the *CD14* exon 2 by sgRNA15. Successful HDR will integrate the SFFV and Kozak sequences in front of exon 2 bypassing exon 1 and intron 1. The complete template sequences are listed in **Table S2**.

Supplementary Figure 8 Inhibition of Aβ phagocytosis by CytoD. **(A)** Workflow of *in vitro* fAβ_1-42_ phagocytosis assay. To block Aβ phagocytic activity, HMC3 cells were pre-treated with CytoD for one hour before fAβ_1-42_ addition and incubated in the drug throughout the assay. The CytoD-treated HMC3 had a rounded morphology. **(B)** Two doses of CytoD were tested. Non-aggregated and aggregated fAβ_1-42_ were also compared. A partial reduction in fAβ_1-42_ phagocytosis was observed at 1 μM CytoD, and a complete inhibition was achieved at 10 μM of CytoD. **(C)** Histogram representations of fAβ_1-42_ phagocytosis.

Supplementary Figure 9 Construction of TREM2 and CD14 double positive cells. **(A)** Two sequential rounds of CRISPR-KI were performed to insert the SFFV promoter at first the *TREM2* gene and then the *CD14* gene. After each CRISPR-KI step, the positive cells were isolated by FACS and expanded. **(B)** Flow cytometry analysis of the double positive cells.

Supplementary Figure 10 Cell viability after Aβ phagocytosis. Representative flow cytometry plots showing the effect of fAβ_1-42_ treatment on HMC3 cell viability at 24 hours of the phagocytosis assay. The number of viable cells (marked by black circles) were determined by Precision beads assay and labeled in the plots.

Supplementary Figure 11 Preparation of apoptotic LN-229 glioblastoma. **(A)** LN-229 cell line is naturally CD40^−^ and CD47^+^. The cell line was modified to express turboGFP by lentiviral transduction. To induce apoptosis, LN-229 cells were treated with increasing concentrations of Paclitaxel (200 nM – 50 μM) for 24 hours. Suspension and adherent LN-229 cells were then separated for Annexin V assay. **(B)** Flow cytometry plots of the suspension LN-229 cells show that the suspension cells were mostly apoptotic. Without Paclitaxel treatment, LN-229 remained adherent to the culture plate, and hence no suspension cell was collected. **(C)** Flow cytometry plots of the adherent LN-229 cells show that the majority of adherent cells were not apoptotic, except at 50 μM of Paclitaxel.

Supplementary Figure 12 Phagocytosis of LN-229 by SIRPα-negative and MERTK-negative mutants cells. Representative flow cytometry plots showing the phagocytosis of live or apoptotic LN-229 cells by HMC3 mutant cells after 24 hours of co-culture.

Supplementary Table 1 The sgRNA list consists the target gene information, guide sequences and PAM. On- and off-target editing scores were predicted *in silico* by CRISPR Design tool on Benchling website. The DNA templates for *in vitro* transcription of sgRNA were synthesized by overlapping PCR using four DNA oligonucleotides as described in Materials and methods. The forward, reverse and bottom oligonucleotides are universal for all sgRNA, but the top oligonucleotides are unique for each sgRNA.

Supplementary Table 2 The complete sequences of HDR templates. The templates for HA-tag insertion at the *RAB11A* and *ACTB* genes were ssDNA ultramers purchased from IDT DNA. The templates for *gfp* insertion at the *RAB11A* and *ACTB* genes were PCR amplified from plasmids pTR143 (Addgene #112012) and AICSDP-15 (Addgene #87425), respectively, using the PCR primers and conditions listed in the table. The templates for SFFV promoter insertion at the *CD14* and *TREM2* gene were constructed as described in Materials and methods. Each template, which consists of the 5’ homology arm, 3’ arm and SFFV promoter sequences, was cloned into a PCR-linearized pUC19 vector by Gibson Assembly. The PCR primers and conditions for the DNA fragments are as listed. The *CD14*-SFFV and *TREM2*-SFFV HDR templates are PCR amplified from plasmids pSL387 and pSL430, respectively.

Supplementary Table 3 Screening for optimal electroporation condition using plasmid pmaxGFP and *CD40*-targeting Cas9 RNP as cargos. Pulse codes for Lonza 4D nucleofector system are listed. The percentages of viability, GFP-positive and CD40-negative cells were determined three days after electroporation by flow cytometry. The top seven codes for plasmid electroporation were further tested for Cas9 RNP electroporation in HMC3 cells. The electroporation of BV-2 cells used a different set of pulse codes. CM158 + SF buffer is recommended by Lonza Nucleofection database, and CA137 + P3 buffer is the best HMC3 condition for cross comparison.

Supplementary Table 4 PCR primers and thermocycler settings for target site amplification for ICE and NGS analyses. The PCR reactions for Sanger sequencing and NGS are as described in the Materials and methods. The NGS barcode sequences are marked in red and blue.
