# Supplementary figures and images for: Interrogation of human microglial phagocytosis by CRISPR genome editing
Source: Front Immunol. 2023 Jul 7;14:1169725. doi: 10.3389/fimmu.2023.1169725 (PMC10360658; doi:10.3389/fimmu.2023.1169725)

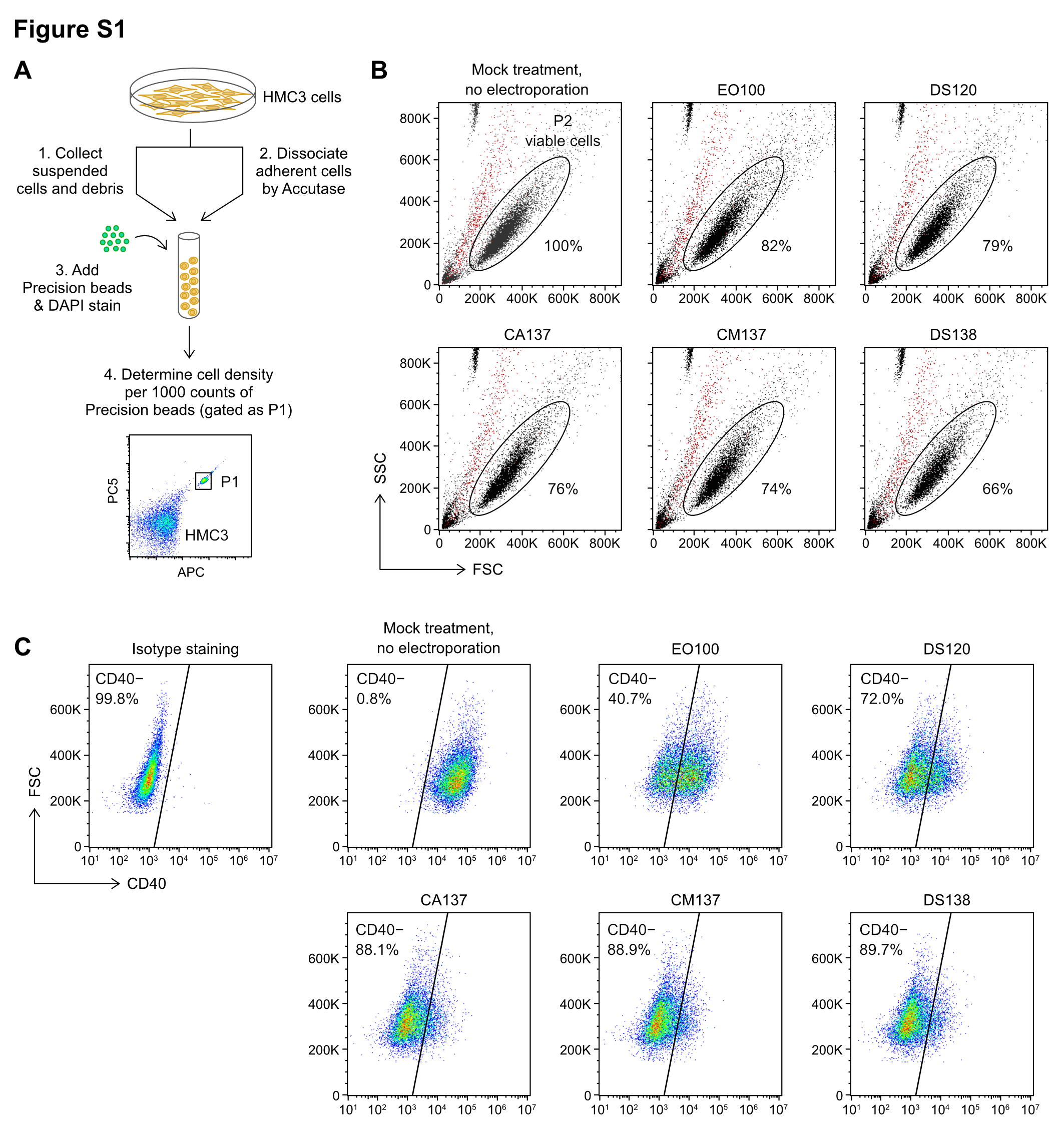

Supplement: Supplementary Figure 1 — Optimization of Cas9 RNP electroporation. (A) Workflow of Precision beads viability assay. Adherent cells, suspended cells and cell debris were all collected and combined in a tube for cell counting by flow cytometry. Precision beads and DAPI dye were then added to the cell suspension. Cell density was determined per 1000 counts of Precision beads (gated as P1). (B) Flow cytometry plots from the Precision beads assay. HMC3 cells were electroporated with Cas9 RNP by five different pulse codes. In the mock treatment, the cells were processed by the same procedure and resuspended in the electroporation buffer, but were not electroporated. The viable HMC3 cells were gated by FSC and side scattering (SSC) as marked by P2 (black circle). Dead cells were DAPI-positive (red). Viable cell counts (%) were normalized to the mock treatment. (C) CD40 KO efficiencies of the five different pulse codes were determined by cell surface staining with anti-CD40 antibodies. [file Image_1.tiff]

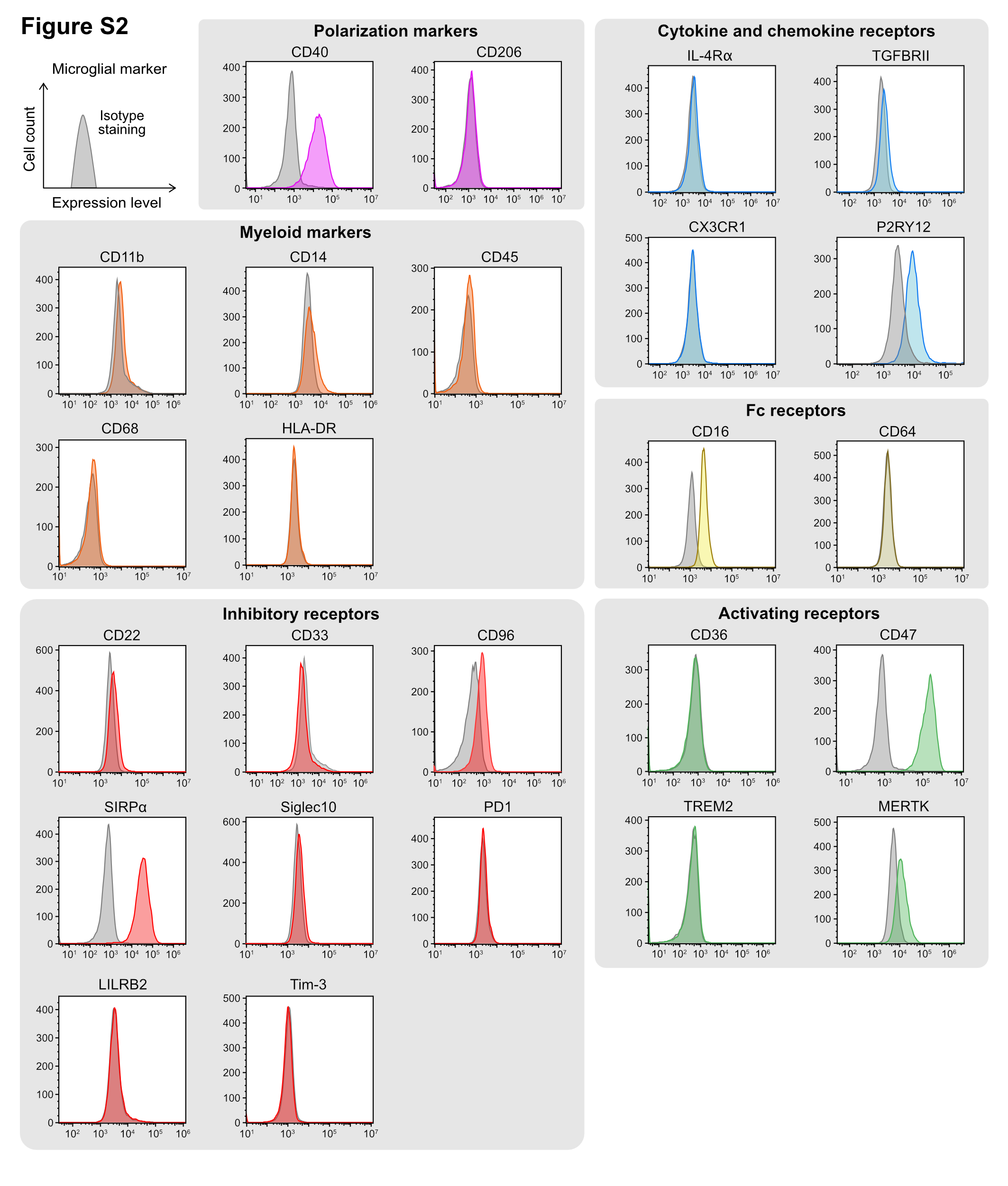

Supplement: Supplementary Figure 2 — Expression profile of surface markers of the parental HMC3 cells. The receptors are categorized by their functions: polarization markers, cytokine and chemokine receptors, myeloid markers, Fc receptors, inhibitory receptors and activating receptors. Isotype control staining is shown in gray peaks, and the marker-specific staining is in colored peaks. The y- and x-axis are cell count and expression level, respectively. [file Image_2.tiff]

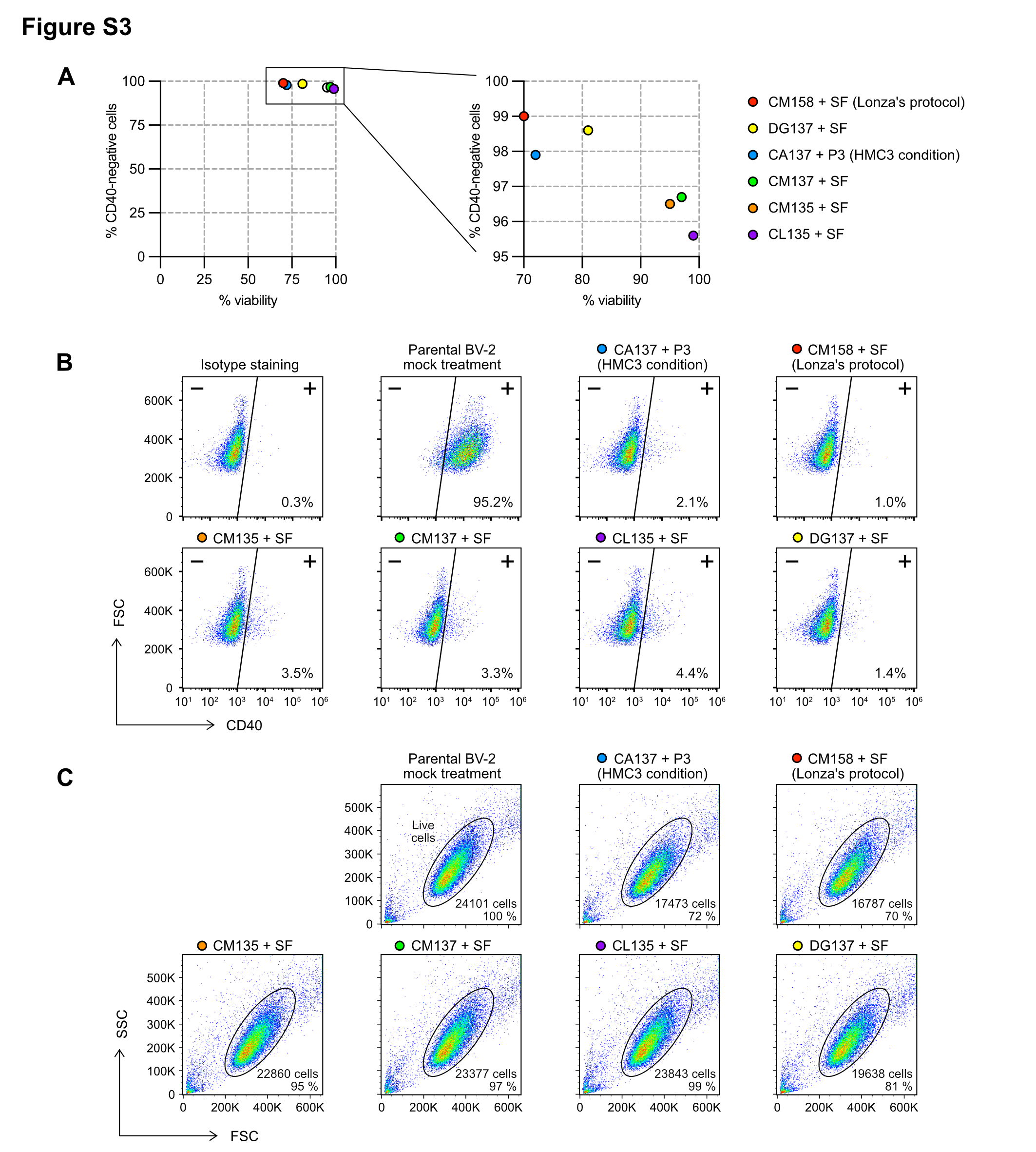

Supplement: Supplementary Figure 3 — Screening of Cas9 RNP electroporation conditions for CD40 KO in BV-2 mouse microglia. (A) Screening was performed using a set of BV-2-specific conditions (color-coded). The % CD40-negative cells and % normalized viability were determined at 72 hours after electroporation. (B) Flow cytometry plots showing the CD40 KO efficiencies of different electroporation conditions. The CD40-negative cells were gated by isotype staining. (C) Flow cytometry plots showing absolute cell counts from Precision beads assay and the % normalized viability under different electroporation conditions. Viable cells were marked by black cycles. [file Image_3.tiff]

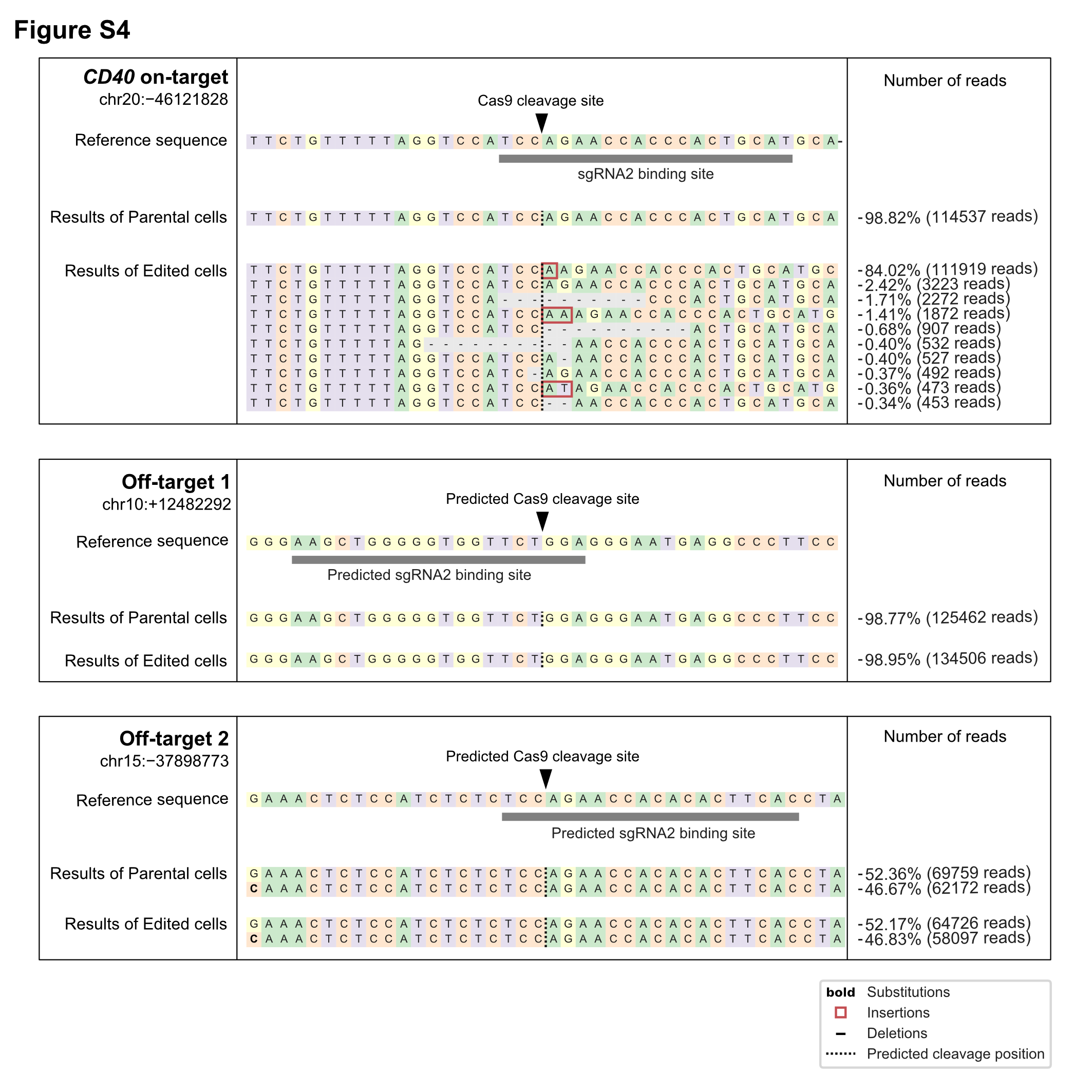

Supplement: Supplementary Figure 4 — Analysis of on- and off-target editing by CD40-targeting Cas9 RNP. The NGS raw data were analyzed and the results were generated by CRISPresso2. The binding positions of sgRNA2 are marked by gray lines. The predicted Cas9 cleavage positions are marked by arrows and dot lines. Red boxes represent base insertions, whereas hyphens represent deletions. Base substitutions are marked by bold letters. [file Image_4.tiff]

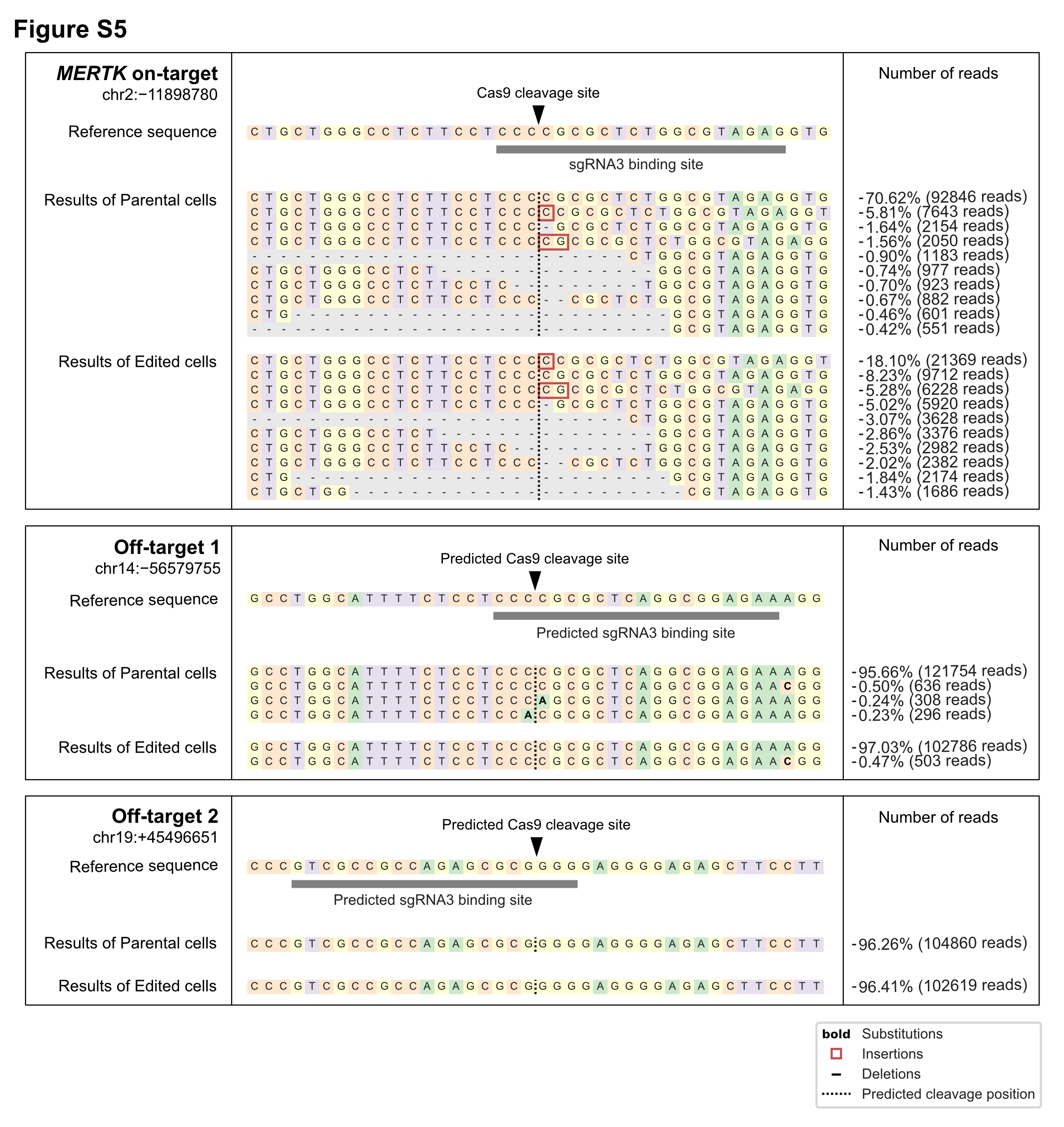

Supplement: Supplementary Figure 5 — Analysis of on- and off-target editing by MERTK-targeting Cas9 RNP. The NGS raw data were analyzed and the results were generated by CRISPresso2. The binding positions of sgRNA3 are marked by gray lines. Red boxes represent base insertions, whereas hyphens represent deletions. Base substitutions are marked by bold letters. The predicted Cas9 cleavage positions are marked by arrows and dot lines. The parental cell population contained about 30% mutations at the MERTK target region. [file Image_5.tiff]

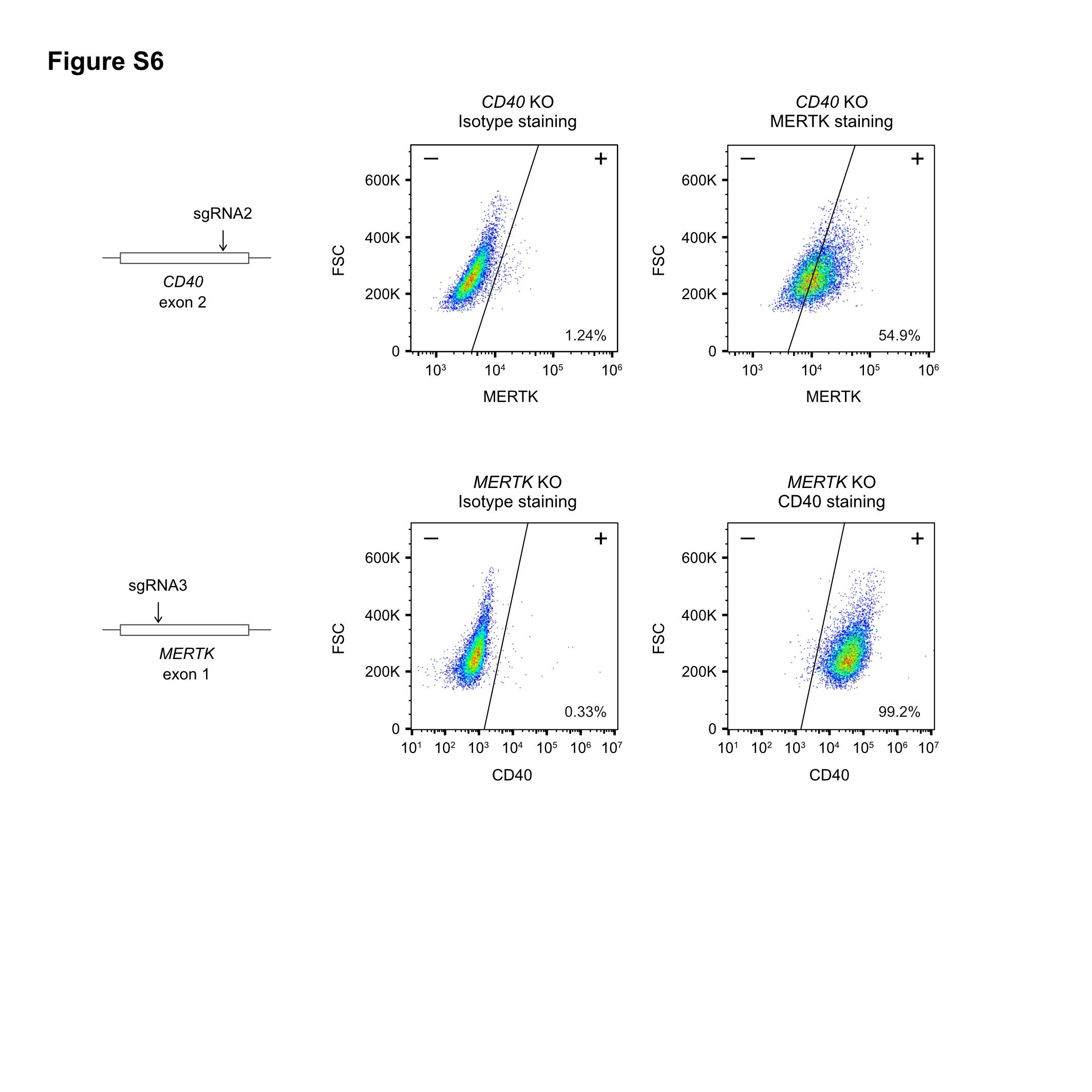

Supplement: Supplementary Figure 6 — Control staining of CD40 and MERTK in the MERTK- and CD40-KO cells. Representative flow cytometry plots showing that the KO of CD40 and MERTK genes do not change the MERTK and CD40 expression, respectively. [file Image_6.tiff]

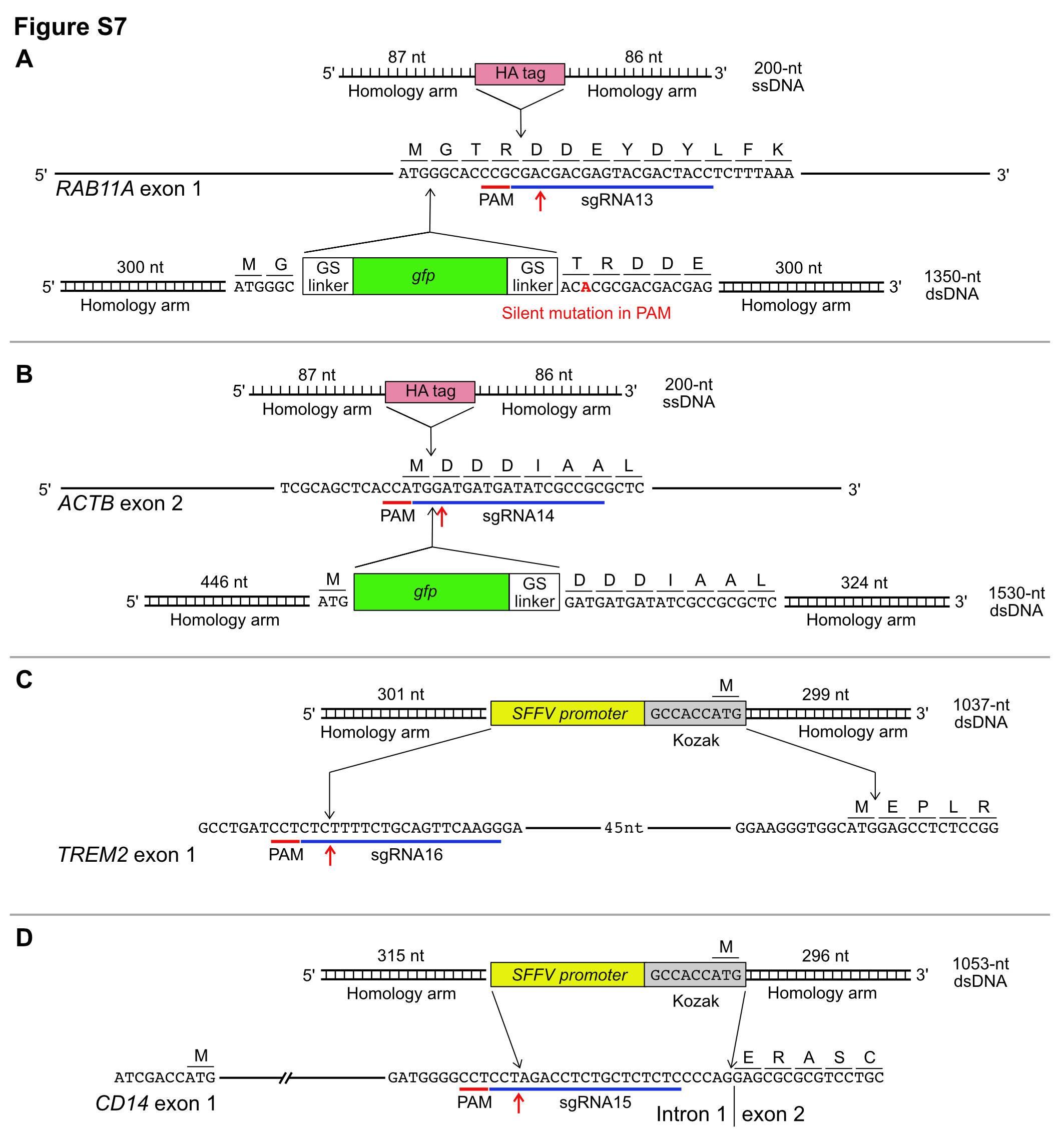

Supplement: Supplementary Figure 7 — Cas9-mediated homology-directed repair (HDR) for the insertion of HA epitope tag, gfp reporter gene and SFFV promoter. (A) The design of HA-tag and gfp templates for insertion at the RAB11A exon 1 region. The HA-tag template is a 200-nt single-stranded synthetic DNA (ssDNA) and the gfp template is a 1350-nt double-stranded DNA (dsDNA) generated by PCR. The sgRNA13 binding site is labeled in blue and the PAM sequence in red. The predicted Cas9 cleavage site is marked by a red arrow. The gfp gene is flanked by glycine and serine (GS) linkers. (B) The design of HA-tag and gfp templates for insertion at the ACTB exon 2 region by sgRNA14. (C) The design of SFFV template for insertion at the TREM2 exon 1 region by sgRNA16. The SFFV template encodes the SFFV promoter and Kozak sequences, flanked by homology arms. Successful HDR will replace the region between sgRNA16 cut site and the start codon with the SFFV and Kozak sequences. (D) The design of SFFV template for insertion at upstream of the CD14 exon 2 by sgRNA15. Successful HDR will integrate the SFFV and Kozak sequences in front of exon 2 bypassing exon 1 and intron 1. The complete template sequences are listed in Table S2 . [file Image_7.tiff]

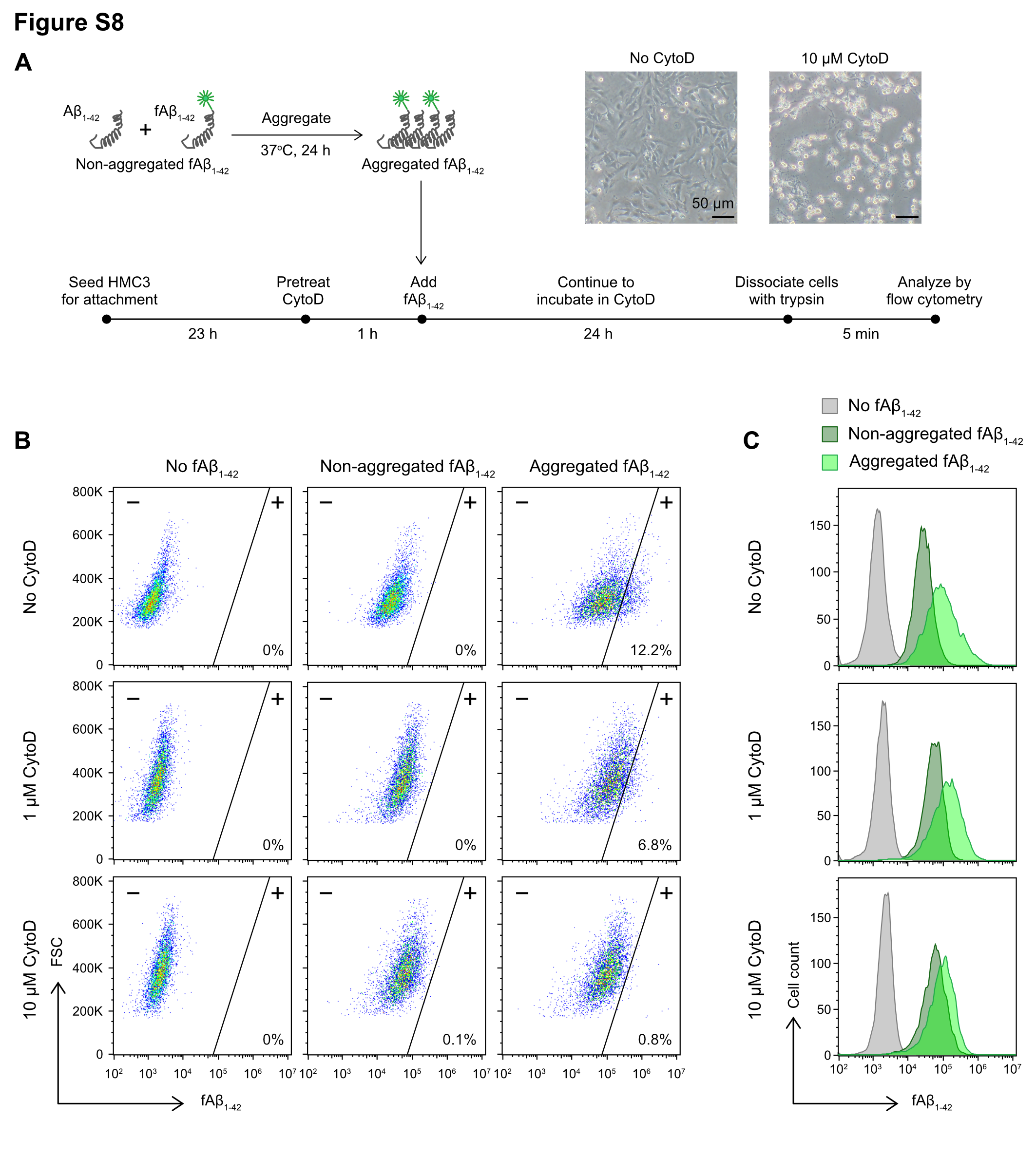

Supplement: Supplementary Figure 8 — Inhibition of Aβ phagocytosis by CytoD. (A) Workflow of in vitro fAβ1-42 phagocytosis assay. To block Aβ phagocytic activity, HMC3 cells were pre-treated with CytoD for one hour before fAβ1-42 addition and incubated in the drug throughout the assay. The CytoD-treated HMC3 had a rounded morphology. (B) Two doses of CytoD were tested. Non-aggregated and aggregated fAβ1-42 were also compared. A partial reduction in fAβ1-42 phagocytosis was observed at 1 μM CytoD, and a complete inhibition was achieved at 10 μM of CytoD. (C) Histogram representations of fAβ1-42 phagocytosis. [file Image_8.tiff]

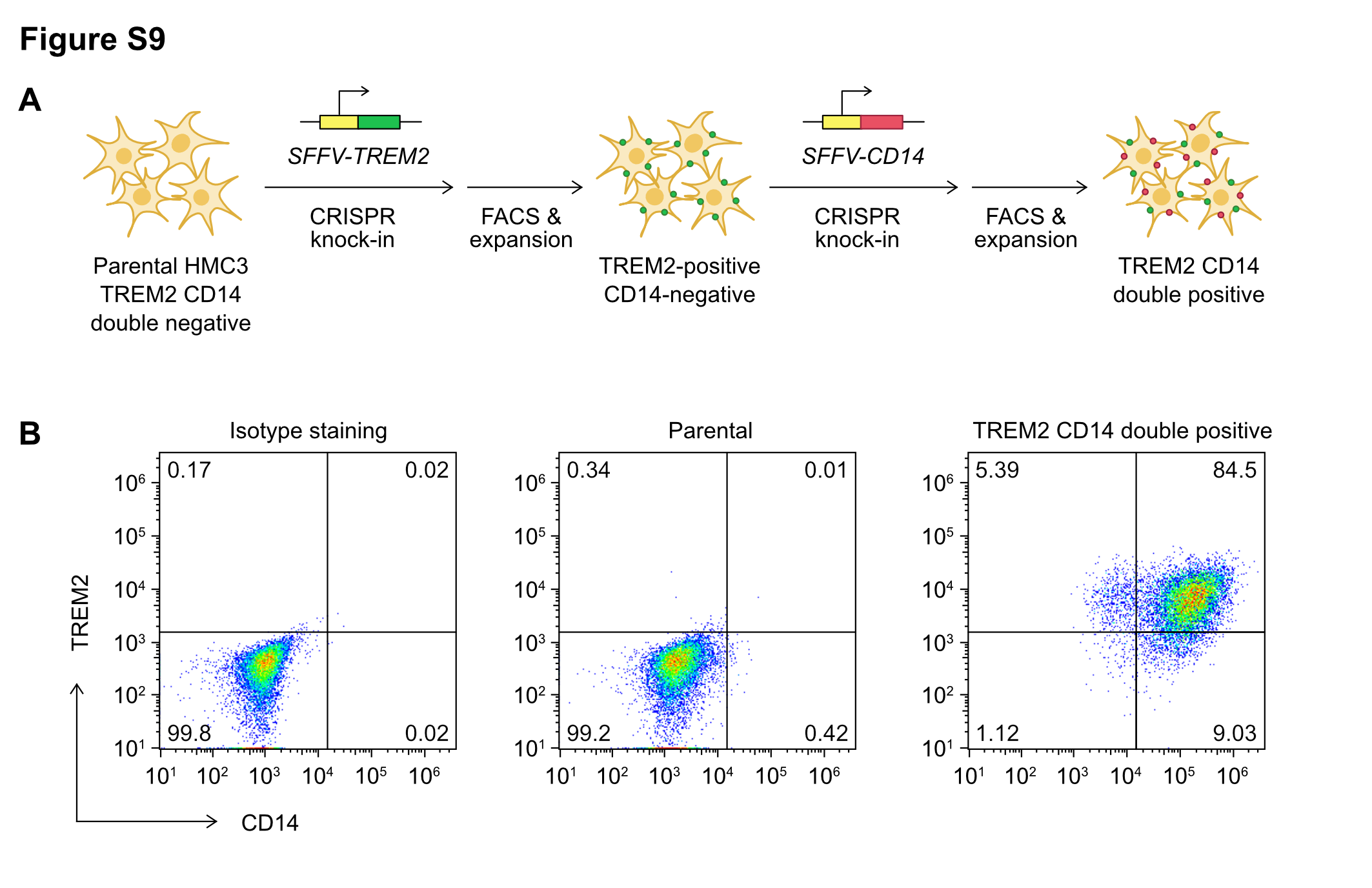

Supplement: Supplementary Figure 9 — Construction of TREM2 and CD14 double positive cells. (A) Two sequential rounds of CRISPR-KI were performed to insert the SFFV promoter at first the TREM2 gene and then the CD14 gene. After each CRISPR-KI step, the positive cells were isolated by FACS and expanded. (B) Flow cytometry analysis of the double positive cells. [file Image_9.tiff]

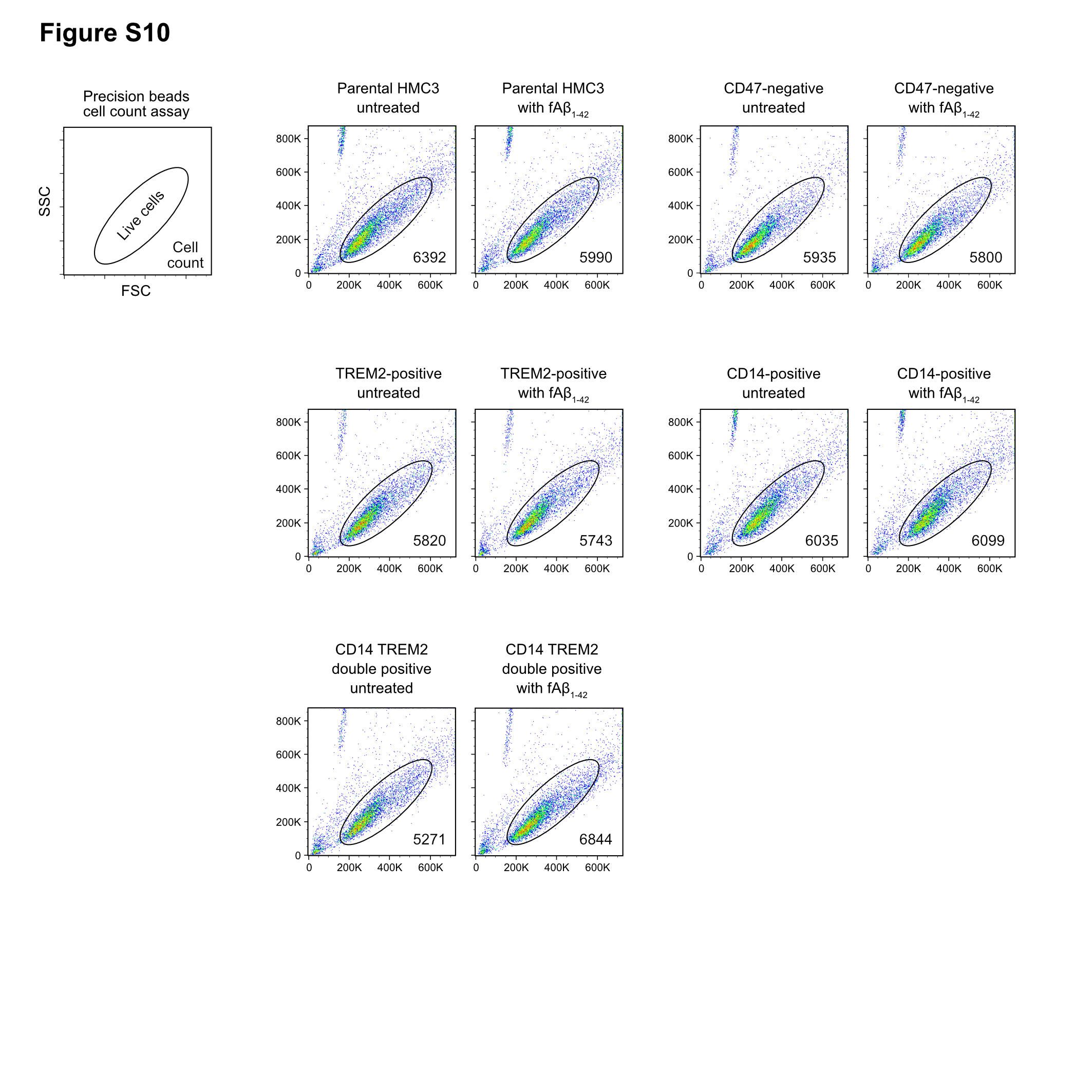

Supplement: Supplementary Figure 10 — Cell viability after Aβ phagocytosis. Representative flow cytometry plots showing the effect of fAβ1-42 treatment on HMC3 cell viability at 24 hours of the phagocytosis assay. The number of viable cells (marked by black circles) were determined by Precision beads assay and labeled in the plots. [file Image_10.tiff]

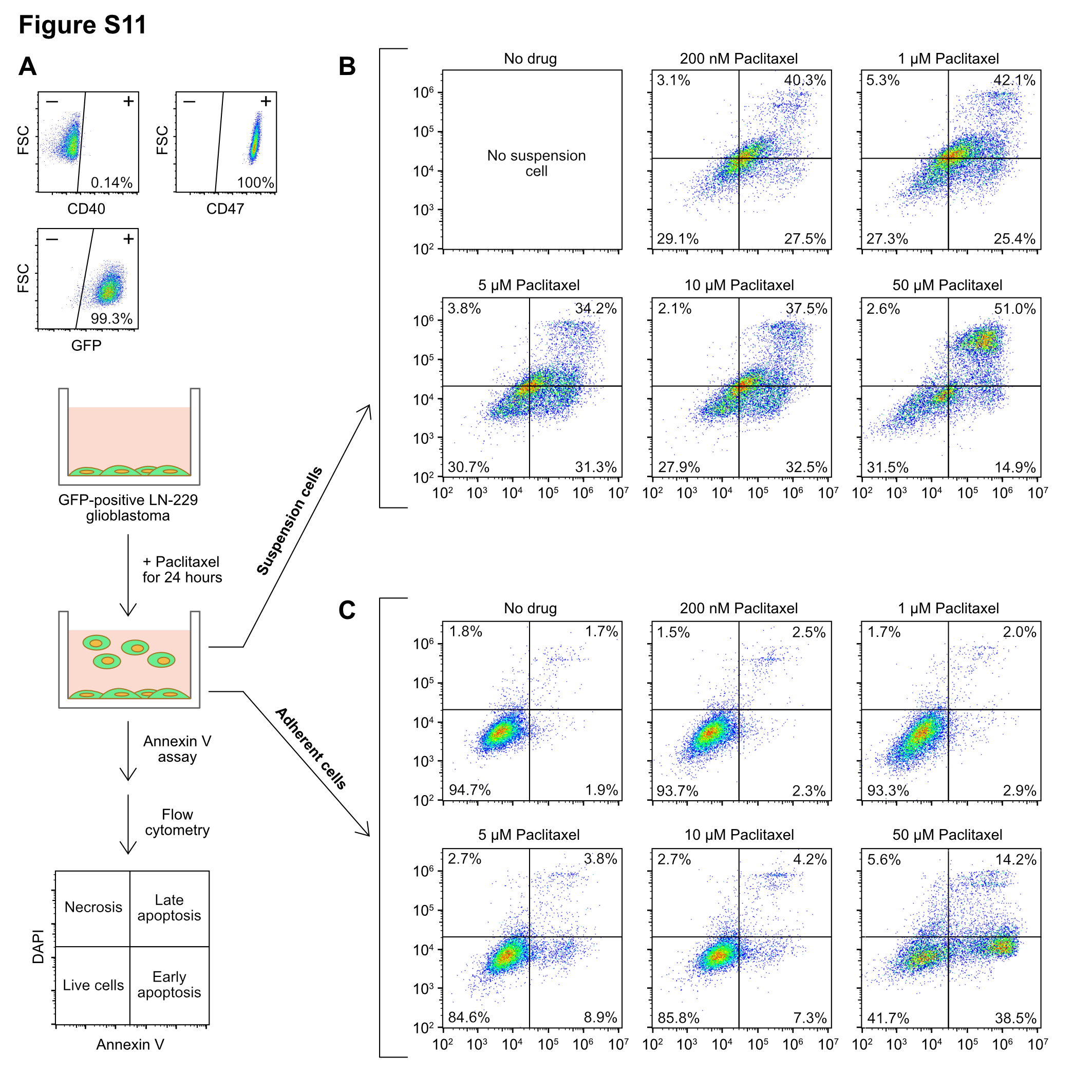

Supplement: Supplementary Figure 11 — Preparation of apoptotic LN-229 glioblastoma. (A) LN-229 cell line is naturally CD40− and CD47+. The cell line was modified to express turboGFP by lentiviral transduction. To induce apoptosis, LN-229 cells were treated with increasing concentrations of Paclitaxel (200 nM – 50 μM) for 24 hours. Suspension and adherent LN-229 cells were then separated for Annexin V assay. (B) Flow cytometry plots of the suspension LN-229 cells show that the suspension cells were mostly apoptotic. Without Paclitaxel treatment, LN-229 remained adherent to the culture plate, and hence no suspension cell was collected. (C) Flow cytometry plots of the adherent LN-229 cells show that the majority of adherent cells were not apoptotic, except at 50 μM of Paclitaxel. [file Image_11.tiff]

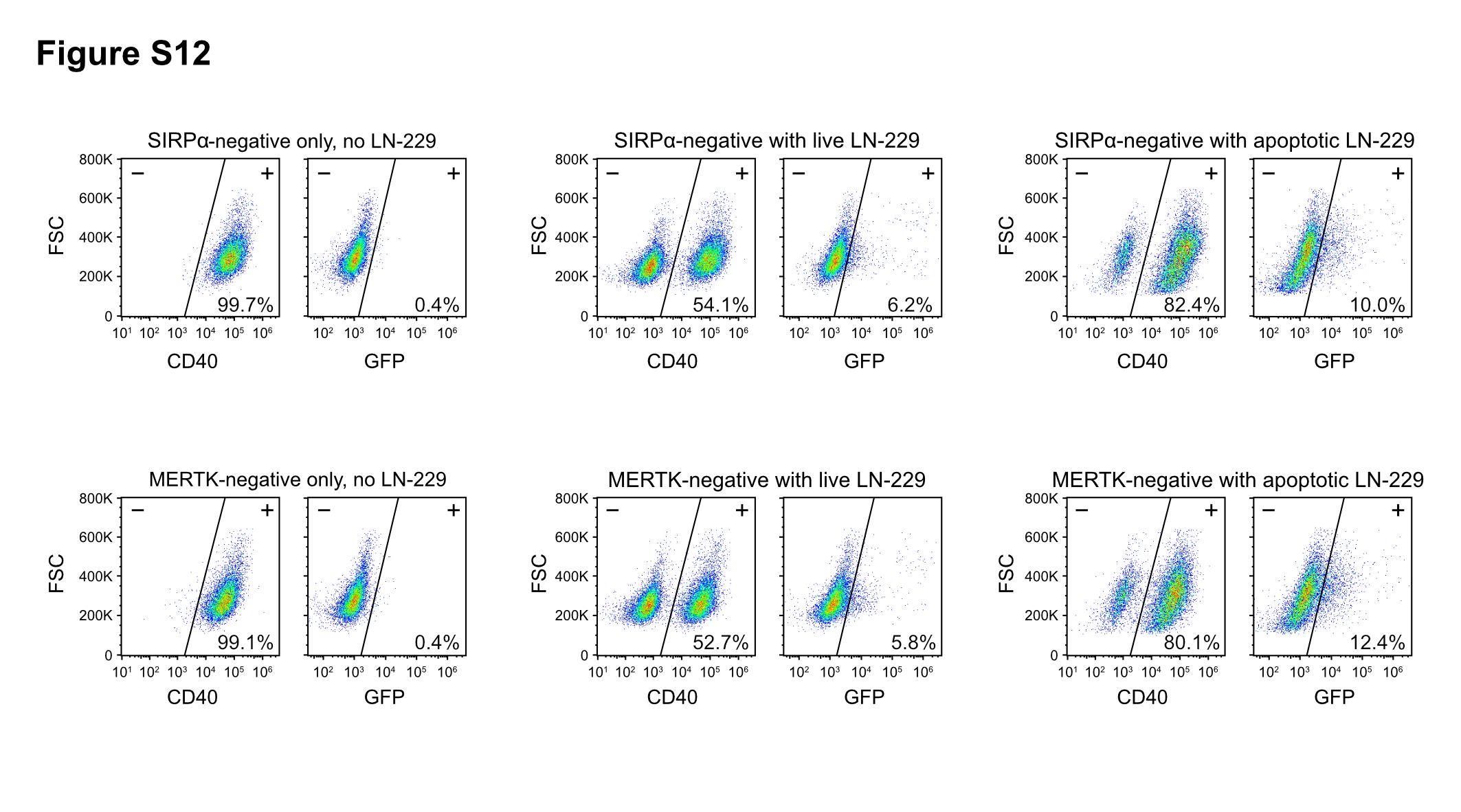

Supplement: Supplementary Figure 12 — Phagocytosis of LN-229 by SIRPα-negative and MERTK-negative mutants cells. Representative flow cytometry plots showing the phagocytosis of live or apoptotic LN-229 cells by HMC3 mutant cells after 24 hours of co-culture. [file Image_12.tiff]
